# Apoptotic signalling targets the post-endocytic sorting machinery of the death receptor Fas/CD95

**DOI:** 10.1038/s41467-019-11025-y

**Published:** 2019-07-15

**Authors:** Shruti Sharma, Antonio Carmona, Agnieszka Skowronek, Fangyan Yu, Mark O. Collins, Sindhu Naik, Claire M. Murzeau, Pei-Li Tseng, Kai S. Erdmann

**Affiliations:** 1 Department of Biomedical Science & Centre of Membrane Interactions and Dynamics, University of Sheffield, Sheffield, S10 2TN UK; 2 Present Address: Department of Radiation Oncology, Dana-Farber Cancer Institute and Brigham and Women’s Hospital, Harvard Medical School, Boston, MA 02115 USA

**Keywords:** Cell death, Lysosomes, Endosomes

## Abstract

Fas plays a major role in regulating ligand-induced apoptosis in many cell types. It is well known that several cancers demonstrate reduced cell surface levels of Fas and thus escape a potential control system via ligand-induced apoptosis, although underlying mechanisms are unclear. Here we report that the endosome associated trafficking regulator 1 (ENTR1), controls cell surface levels of Fas and Fas-mediated apoptotic signalling. ENTR1 regulates, via binding to the coiled coil domain protein Dysbindin, the delivery of Fas from endosomes to lysosomes thereby controlling termination of Fas signal transduction. We demonstrate that ENTR1 is cleaved during Fas-induced apoptosis in a caspase-dependent manner revealing an unexpected interplay of apoptotic signalling and regulation of endolysosomal trafficking resulting in a positive feedback signalling-loop. Our data provide insights into the molecular mechanism of Fas post-endocytic trafficking and signalling, opening possible explanations on how cancer cells regulate cell surface levels of death receptors.

## Abstract (web summary)

Fas is a death receptor that regulates apoptosis in many cell types and is downregulated on the cell surface in many cancers. Here, Sharma et al. show that endosome associated trafficking regulator ENTR1 regulates delivery of Fas to lysosomes, thereby controlling its degradation and signalling.

## Introduction

Fas (CD95/Apo-1) is a member of the tumour necrosis receptor family and the prototype of a death receptor, which can cause ligand-induced apoptosis via activation of a protease cascade^1^. Fas exists as a preassociated trimer at the plasma membrane^2,3^. Ligand binding to Fas induces a conformational change allowing its cytoplasmic death domain to engage with other proteins, which leads to the formation of the so-called death-inducing signalling complex (DISC)^1,4^. Key to the formation of DISC is the association of Fas with the Fas associated death domain protein FADD, which itself is associated with the proteases procaspase-8 and 10^1,4–6^. Procaspase-8/10 are cleaved and activated upon activation of Fas in an autoproteolytic process initiating a protease cascade involving other caspases (e.g. 3 or 6) further downstream leading eventually to apoptosis^7–9^. It has been shown that membrane trafficking of the death receptor plays an important role in Fas apoptotic signalling and that efficient formation of DISC takes place at the level of endosomes and thus requires the endocytosis of Fas^6^. Furthermore, depending on cell type and context, non-apoptotic signalling pathways such as the activation of transcription factor NF- κB can also be activated upon ligand binding to Fas^10^.

Physiologically, Fas plays a crucial role in the regulation of peripheral immune tolerance as well as in lymphoid homeostasis^11^. However, it also plays an important role in tumour progression. The role of Fas in cancer is complex and anti-tumour activities as well as tumour-promoting activities have been reported for the Fas/Fas-ligand system^11^. It is known that during tumour progression cancer cells undergo a number of changes. One of the early changes observed is increased resistance to apoptosis, which allows cancer cells to escape important control mechanisms^12^. In line with this is the observation that many cancer cells acquire resistance to Fas-induced apoptosis. This resistance can be achieved either by upregulation of anti-apoptotic proteins^13^, by acquiring mutations in the Fas gene itself^14^ or by downregulation of Fas^15^. Downregulation of Fas takes place by two main mechanisms, which are not mutually exclusive. One possibility is the downregulation of total Fas protein levels, another possibility is to regulate the availability of the receptor at the cell surface^16–20^. Thus, molecular mechanisms and molecular players regulating cell surface levels of Fas are of great interest. One such molecular player is the protein tyrosine phosphatase PTPN13 (also known as FAP-1, PTPL1 or PTP-BAS), which has been identified as a negative regulator of Fas cell surface levels and Fas-induced apoptosis^16,17,21,22^. PTPN13 interacts directly with Fas^21,23^ and high expression levels of PTPN13 inversely correlate with Fas cell surface levels^16–18,24^. The microRNA mir-200c, which is often downregulated in early stages of cancer, confers sensitivity to Fas-induced apoptosis via targeting PTPN13^25^. We have recently shown that PTPN13 forms a complex with the endosome associated trafficking regulator ENTR1, previously named SDCCAG3 (serologically defined colon cancer antigen-3)^26^. ENTR1 is an endosomal protein localised to early and recycling endosomes^26^, which can associate with the retromer component VPS35, and which plays a role in cilia formation^27–29^. ENTR1 was originally identified as an antigen in serum derived from colon cancer patients^30^. Subsequently, it was identified in a screen for proteins regulating sensitivity to tumour necrosis factor induced apoptosis^31^, however the underlying mechanism for this remained unclear.

Here we show that ENTR1 is a negative regulator of Fas cell surface levels and Fas-induced apoptosis. We demonstrate that ENTR1 is important for endolysosomal sorting of Fas. Moreover, we show that ENTR1 is cleaved during Fas-induced apoptosis revealing a positive feedback loop in apoptotic signalling. Our data provide mechanistic insights into how membrane trafficking regulates sensitivity to Fas-induced apoptosis.

## Results

### Depletion of ENTR1 increases Fas cell surface levels

**Fig. 1:**
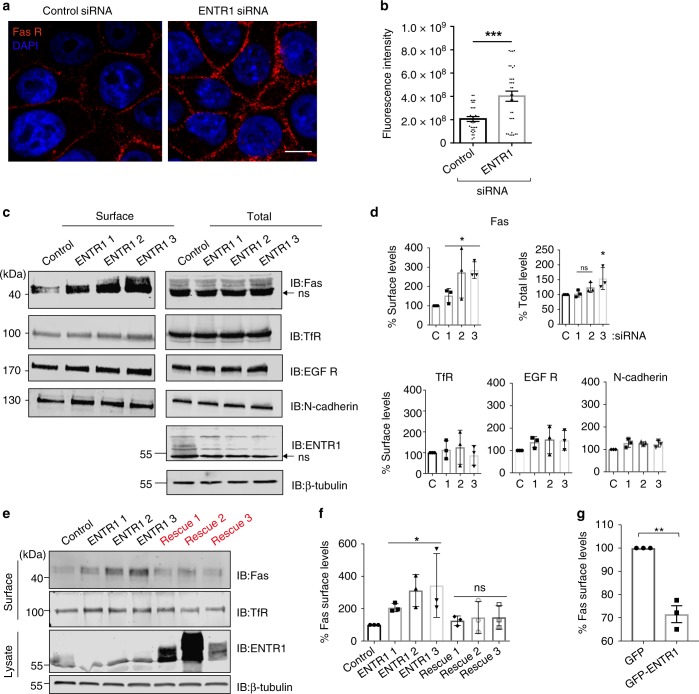
Surface levels of Fas receptors are increased upon silencing ENTR1. **a** Immunofluorescence analysis of cell surface levels of Fas receptors upon ENTR1 no.2 and control knock-down in HeLa cells. Non-permeabilised HeLa cells were stained with Anti-Fas (CH-11) followed by Alexa 594 conjugated secondary antibody staining. Images were obtained using a confocal microscope. Scale bar represents 5 µm. **b** Quantitative analysis of CTCF (corrected total cell fluorescence) represented as scatter plot with bars. Data were analysed by unpaired Student’s *t*-test, *n* = 3, **(*p* < 0.05), *p* value = 0.0073, error bars represent ± s.e.m. **c** Immunoblot analysis of biotinylated cell surface fraction and total lysate of HeLa cells treated with three independent siRNA against ENTR1 and control. Fas receptors were detected using anti-Fas (C-20) antibody; endogenous levels of other mentioned receptors were detected using their specific antibodies; the arrow indicates non-specific (ns) bands; ß-tubulin was used as a loading control. **d** Quantitative analysis of cell surface levels of the indicated receptors represented as bar graphs. On x-axis, ‘C’ stands for control siRNA and 1–3 stands for the three siRNAs against ENTR1. The y-axis represents relative abundance of receptors expressed as a percentage when control is 100%. The quantification shows mean of at least three independent experiments (*n* = 3), error bars represent ± s.e.m; one-way ANOVA test, *p*-value; **< 0.05; *p* = 0.0340 for Fas. **e** Immunoblot analysis of surface levels of Fas upon transfection with ENTR1 siRNA alone or co-transfection with siRNA and siRNA resistant ENTR1 constructs (labelled as Rescue 1–3) in HeLa cells. Uncropped blots are shown in supplementary Fig. 11. **f** Quantification of surface levels of Fas receptors normalised to ß-tubulin and expressed as a percentage where control is 100%. Data was collected form three independent experiments (*n* = 3, One way ANOVA, *p < 0.05, *p* = 0.045, ns-not significant). Error bars represent ± s.e.m. **g** Flow cytometry analysis of surface abundance of Fas in HeLa cells upon GFP-ENTR1 overexpression. Quantification of the flow cytometry analysis of Fas levels expressed as a percentage where GFP-transfected HeLa cells control is 100%. Bar graphs represent the mean of the Alexa Fluor 488 nm median intensities from three independent experiments (*n* = 3), student *t*-test, error bars represent ± s.e.m

Recently we identified ENTR1 as a binding partner for the protein tyrosine phosphatase PTPN13^26^. PTPN13 is a known inhibitor of Fas-induced apoptosis and negatively regulates Fas cell surface levels by an unknown mechanism^16^. Given the tight interaction of ENTR1 with PTPN13 we tested whether depletion of ENTR1 expression levels also changes Fas cell surface levels. To address this question, we analysed Fas cell surface levels in HeLa cells using immunofluorescence microscopy upon siRNA mediated depletion of ENTR1. We observed a significant increase in immunofluorescence of Fas at the plasma membrane (Fig. 1a, b). Furthermore, we confirmed increased Fas surface levels upon ENTR1 depletion biochemically using surface biotinylation followed by anti-Fas western blotting. The increase was specific for Fas because, by contrast, we did not observe such changes for other housekeeping and cell surface signalling receptors such as the transferrin receptor, the epidermal growth factor (EGF) receptor or N-Cadherin (Fig. 1c, d). Furthermore, transfection of the siRNAs did not have any effect on the mRNA levels of Fas as measured by quantitative PCR (Supplementary Fig. 1). The increase in surface Fas was reflected in a trend towards increased levels of total Fas, which was significant for siRNA 3 (Fig. 1d). Next, we used fluorescence flow cytometry to measure Fas cell surface levels upon ENTR1 depletion and confirmed our findings above (Supplementary Fig. 2). Further experiments showed that increased Fas surface expression levels could be returned to control levels upon transfection of the corresponding siRNA resistant ENTR1 expression construct for all three siRNAs applied (Fig. 1e, f). We also confirmed our findings in another cell line (the human colon carcinoma cell line HCT116). In particular we observed here a significant increase in cell surface and total Fas expression levels following ENTR1 siRNA treatment (Supplementary Fig. 3a–d). Our data together strongly suggest that increased cell surface levels of Fas depend on decreased ENTR1 expression levels. In line with this is also our finding that increasing ENTR1 expression levels by transfecting a GFP-ENTR1 expression construct correlates with decreasing Fas cell surface levels in HeLa cells (Fig. 1g).

### Regulation of Fas cell surface levels requires PTPN13

**Fig. 2:**
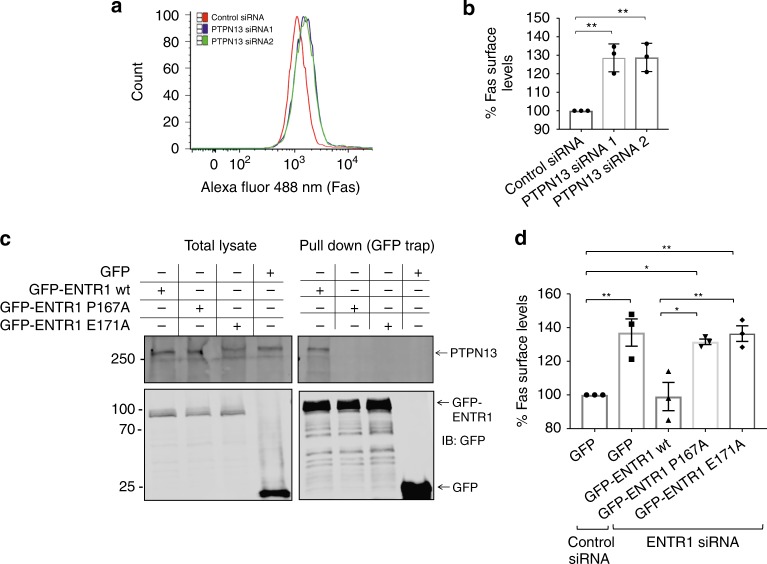
ENTR1 requires binding to PTPN13 to regulate Fas cell surface levels. **a** Flow cytometry analysis of surface abundance of Fas in HeLa cells upon PTPN13 knock-down. Shift of the peaks towards the right in all the samples as compared to the control indicates increased Alexa 488 intensity. **b** Quantification of the flow cytometry analysis of Fas receptor levels expressed as a percentage relative control siRNA transfected cells. Bar graphs represent the mean of the Alexa Fluor 488 nm median intensities (*n* = 3), three independent experiments, one way ANOVA. **c** Interaction of wild-type and mutant ENTR1 with endogenous PTPN13. HeLa cells were transfected with the indicated GFP-tagged ENTR1 constructs followed by GFP-trap and western blotting. **d** Flow cytometry analysis of Fas surface levels upon ENTR1 knock-down and rescue with aforementioned ENTR1 constructs. Bar graphs represent the mean of the Alexa Fluor 633 nm median intensities. Data was collected from three independent experiments (*n* = 3), One-way ANOVA was performed, **p < 0.01, error bars represent ± SEM

PTPN13 binds directly to Fas via its PDZ2 domain and is a known negative regulator of Fas cell surface expression^16,21^; using two different siRNAs we could confirm that lowering PTPN13 levels correlates with increased Fas cell surface expression also in HeLa cells (Fig. 2a, b). In order to test whether interaction with PTPN13 is important for ENTR1 to regulate Fas cell surface levels we tested two ENTR1 point mutations, P167A and E171A, which we had previously identified to abolish interaction with the FERM domain of PTPN13^26^. Here we demonstrate using co-immunoprecipitation experiments that these point mutations also abolish interaction with full-length PTPN13 (Fig. 2c). In order to test whether interaction with PTPN13 is important for ENTR1 function, we performed rescue experiments measuring Fas cell surface levels. HeLa cells were depleted of endogenous ENTR1 and transfected with siRNA resistant expression constructs for wild-type ENTR1, ENTR1-P167 or ENTR1-E171A. Whereas transfection with wild-type ENTR-1 lead to a full rescue of Fas cell surface expression levels, we could not observe any rescue effect with the mutant ENTR1 constructs (Fig. 2d). This result strongly suggests that the formation of a PTPN13/ENTR1 protein complex is required to regulate Fas surface expression levels.

### Fas co-localises with ENTR1 and PTPN13

**Fig. 3:**
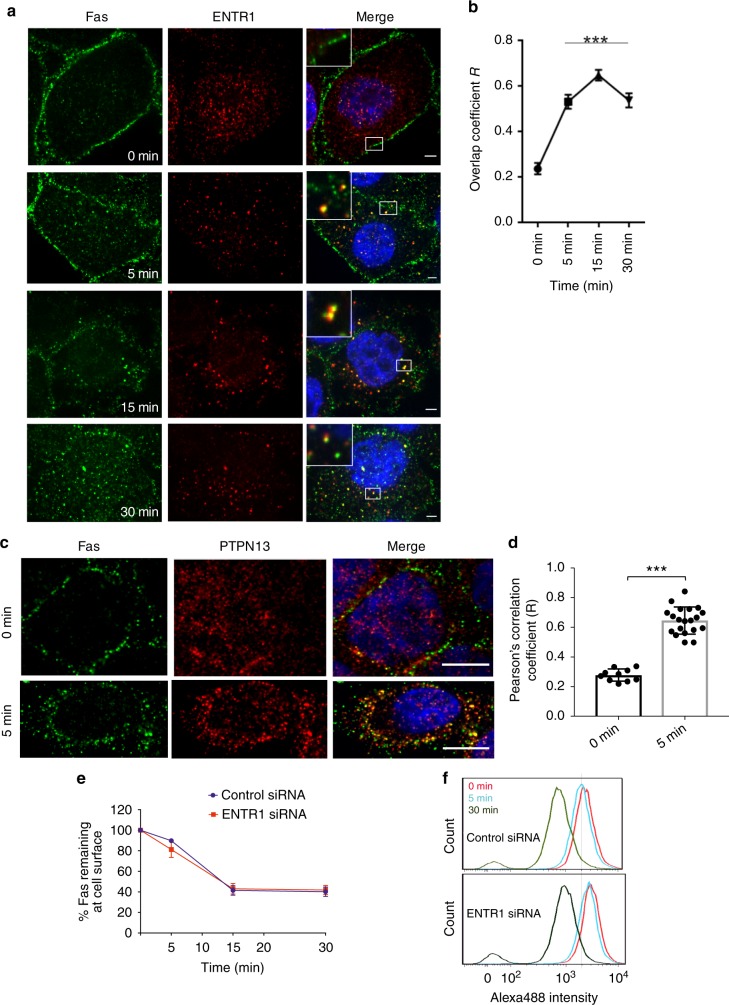
Internalised Fas co-localises with ENTR1 and PTPN13 positive endosomes. **a** Immunofluorescence analyses of co-localisation between Fas (green) and ENTR1 (red). Fas was surface labelled with anti-Fas (CH-11) antibody (1 µg/ml) and were allowed to internalise for the indicated time points. Cropped inset panels show examples of non-existent (0 min) or existent co-localised particles. Images were acquired from a confocal microscope. Scale bars represent 5 µm. **b** Quantification of co-localisation between internalised FasR and ENTR1 (calculated using Mander’s overlap coefficient R). Data was analysed from five cells (for 0, and 30 min) and eight cells (for 5, and 15 min time points) using Tukey’s multiple comparison tests, *p < 0.05, ***p < 0.01. **c** Immunofluorescence analysis of co-localisation between PTPN13 and Fas receptors. HeLa cells were stimulated with 1 μg/ml of anti-Fas (CH-11) antibody (green) for the indicated time points. Cells were fixed and stained for PTPN13 (red). Scale bar represents 5 μm. **d** Quantification of co-localisation observed in **c**. Pearson’s correlation coefficient was calculated using JACoP tool in image J. Data represents average from three independent experiments (*N* = 3) form at least (*n* > 30 cells). Student’s *t*-test, **p < 0.05, error bars represent ± s.e.m. **e** Flow cytometry analysis of internalisation of Fas induced upon activation with anti-Fas (CH-11) antibody (1 µg/ml) for various time points. Relative amount of Fas receptors remaining on the cell surface was expressed as a percentage where 0 min was 100% in control treated HeLa cells. Data was collected from three independent experiments (*n* = 3) and analysed using Tukey’s multiple comparisons tests, error bars represent ± s.e.m. **f** Representative histograms showing surface population of Fas after 0, 5 or 30 min of internalisation

In order to understand the molecular mechanism by which depletion of ENTR1 elevates Fas levels, we first analysed whether both proteins co-localise within the cell. We have previously demonstrated that ENTR1 localises to early/recycling endosomes suggesting a potential role in membrane trafficking^26^. We could not observe significant co-localisation of Fas and ENTR1 under non-stimulated conditions, where most Fas appeared to be localised to the plasma membrane and ENTR1 localised to intracellular punctae. However, following stimulation of Fas with the agonistic antibody CH11 we observed co-localisation of ENTR1 and Fas in punctate structures as early as five minutes after stimulation in line with a co-localisation of both proteins at endosomes (Fig. 3a, b). We also observed co-localisation of Fas and PTPN13 in early endosome antigen-1 positive punctates after stimulation of Fas (Fig. 3c, d, Supplementary Fig. 4). Interestingly, whereas ENTR1 appears to be constitutively localised to endosomes we only observed PTPN13 at endosomes after Fas stimulation. Together this suggests that Fas co-localises with ENTR1 and PTPN13 in early endosomes.

### Depletion of ENTR1 correlates with delayed Fas degradation

**Fig. 4:**
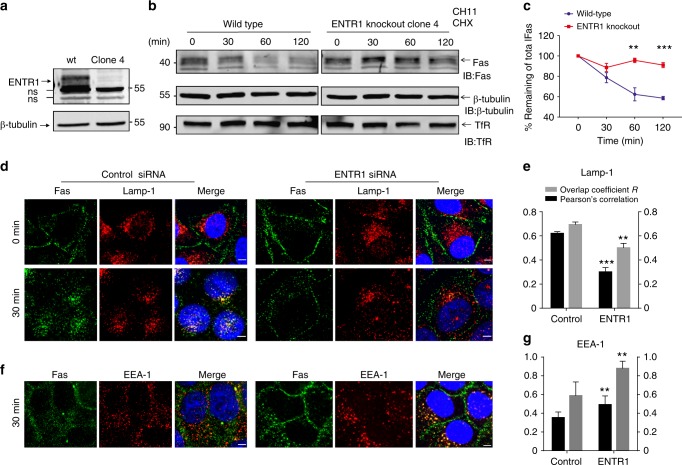
ENTR1 knock-out cells show delayed degradation of Fas. **a** Western blot validation of ENTR1 knock-out in HCT-116 cell line generated by CRISPR-Cas9. **b** Immunoblot analysis of the kinetics of degradation of endogenous Fas activated by agonistic anti-Fas (CH-11) antibody (500 ng/ml) in the presence of cycloheximide (150 µg/ml) for the indicated time points. ß-tubulin was used as a loading control. **c** Relative receptor abundance expressed as a percentage where 0 min is 100% in wild-type or ENTR1 knock-out HCT-116 cells, respectively. Data was collected from three independent experiments (*n* = 3) and analysed by multiple comparisons test, *p < 0.05, error bars represent ± SEM. **d**, **f** Immunofluorescence analysis of sub-cellular localisation of Fas receptors in HeLa cells upon stimulation with agonistic anti-Fas (CH-11) antibody (1 µg/ml) in the presence of Leupeptin (100 nM) for the indicated time points. Upon internalisation, Fas receptors (green) co-localised with Lamp-1 positive vesicles (red) in control siRNA treated cells in contrast with ENTR1 no.2 siRNA treated cells after 30 min of antibody chase. Additionally, Fas receptors in control cells (green) did not co-localise with EEA-1 (red) substantially as compared to the ENTR1 depleted cells after 30 min of antibody chase. Images were acquired using a confocal microscope. Scale bar represents 5 µm. **e**, **g** Quantification of the extent of correlation (Pearson’s correlation coefficient, PCC) and co-localisation (Mander’s overlap coefficient R) between Fas receptors and endosomal markers such as Lamp-1 and EEA-1. Co-localisation analysis was performed on confocal cross-sections using JACoP (Just another co-localisation plugin) in ImageJ. Data was collected from three independent experiments *n* = 3, *t*-test, **p < 0.01, ***p < 0.001, error bars represent ± s.e.m

Increased cell surface expression levels of Fas could be due to impaired endocytosis upon ENTR1 depletion. To test this possibility HeLa cells were transfected with siRNA targeting ENTR1 or control siRNA and endocytosis was monitored by fluorescence flow cytometry after addition of CH11. There was no difference in the kinetics of Fas endocytosis (measured as % of remaining Fas at the cell surface after stimulation) in ENTR1 depleted cells versus non-depleted cells suggesting that impaired endocytosis does not account for the increase in cell surface levels upon ENTR1 knock-down (Fig. 3e, f). In order to investigate any changes in degradation kinetics of Fas we generated HCT116 cells lacking ENTR1 using the CRISPR/Cas9 system (Fig. 4a, Supplementary Fig. 8c). Wild-type and ENTR1 knock-out cells were stimulated with CH11 for different times and the amount of Fas was measured by Western-blotting. Whereas there was a clear decline of Fas expression levels over time in wild-type HCT116 cells there was only a minor degradation of Fas observable in cells lacking expression of ENTR1 (Fig. 4b, c). Furthermore, Fas degradation could be blocked by leupeptin indicating lysosomal degradation (Supplementary Fig. 5c). Similar results were also found by downregulating ENTR1 using siRNA (see supplementary Fig. 5a, b). In contrast ENTR1 KO appears not to affect EGF receptor degradation (Supplementary Fig. 7f, g). In summary our data show that decreased ENTR1 expression levels correlate with impaired Fas degradation.

### Depletion of ENTR1 impaires transport of Fas to lysosomes

Our observation that Fas degradation is delayed in ENTR1 KO cells together with our finding that Fas and ENTR1 co-localise at endosomes upon activation prompted us to investigate the endolysosomal transport route and kinetics of Fas in more detail. Under control conditions Fas is efficiently transported to lysosomes within 30 min after Fas activation as demonstrated by co-localisation with the late endosomal/lysosomal marker Lamp-1. However, in ENTR1 depleted cells, transport to lysosomes was significantly impaired, which is reflected in a reduced overlap coefficient and reduced Pearson´s correlation coefficient (Fig. 4d, e). Interestingly, we made a similar observation for non-stimulated Fas by performing an antibody-feeding experiment using the non-agonistic anti-Fas antibody DX-2 (Supplementary Fig. 6). These observations open the possibility that transport of Fas from early endosomes to lysosomes might be impaired. This was indeed the case as we observed a decrease in co-localisation with Lamp-1 whereas there was a concomitant significant increase in co-localisation of Fas with early endosome marker EEA1 in ENTR1 depleted cells compared to control siRNA transfected cells after 30 min of Fas activation (Fig. 4f, g). In summary our data are in line with an impairment of Fas to transit properly from the early endosomal compartment to the lysosomal compartment.

### ENTR1 impairs sorting of Fas into intraluminal vesicles

**Fig. 5:**
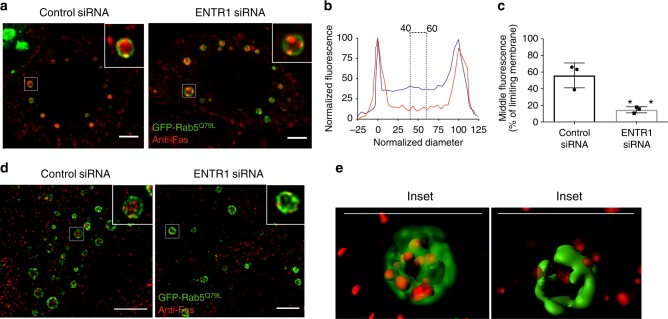
Depletion of ENTR1 delays sorting of Fas into intraluminal vesicles. **a** Immunofluorescence of Fas in cells transfected with constitutively active Rab5 Q79L. Control or ENTR1 no.3 siRNA treated HeLa cells were transfected with Rab5 Q79L-GFP and stimulated with anti-Fas (CH-11) antibody (1 µg/ml) for 1 h in the presence of leupeptin (100 nM). Cells were fixed and observed by a confocal microscope. Insets show representative expanded endosomes. **b** Example of line scale analysis used for quantifying Fas localised to intraluminal vesicles. The normalised diameter represents the diameter of the endosome, where 0 and 100 correspond to the pixel distances with the highest and second highest pixel intensities, representing the limiting membranes of the endosomes. Blue and red traces represent the normalised fluorescence pixel intensity measured across the endosomes in control and ENTR1 depleted cells, respectively, with the maximum pixel intensity across the line normalised to 100. Region covered by dotted line shows the normalised fluorescence values of pixels from 40–60% of the normalised diameter that were used to determine the mean intraluminal fluorescence for each endosome. **c** Graphical representation of the compiled results of the line scale analysis for Fas. Middle (40–60%) fluorescence value expressed as a percentage of the limiting membrane (normalised diameter). Unpaired *t*-test, *n* > 50 endosomes from three independent experiments, **p < 0.01, *p* = 0.0075, error bars represents ± s.e.m. **d** Immunofluorescence analysis of intraluminal sorting of Fas in control or ENTR1 no.3 siRNA treated HeLa cells transfected with constitutively active Rab5 Q79L using 3D-Structured illumination microscopy. **e** Three-dimensional modelling of endosomes highlighted by the insets in **d**. Scale bars represent 5 µm (**a**, **d**, **e**)

Following internalisation, the canonical endolysosomal route for transmembrane proteins is their transfer from the limiting endosomal membrane into intraluminal vesicles of multivesicular bodies. For many receptor-mediated signalling systems this is the point where signal transduction is irreversibly shut off^32,33^. In order to test whether this step might be affected by ENTR1 depletion we transfected HeLa cells with constitutively active GFP-Rab5, which promotes the formation of enlarged endosomes^34^ and thus facilitates the detection of Fas in the limiting membrane and within the lumen of multivesicular bodies. This method has been used previously to analyse transfer of other cargos from the limiting membrane into the lumen of multivesicular bodies^35^. HeLa cells were treated with control or ENTR1 siRNA for 72 h. Fas receptors were stimulated with CH11 and examined for their intracellular localisation by confocal microscopy. After 2 h of stimulation, Fas was present mostly inside the lumen of enlarged vesicles positive for Fas under control conditions. In contrast, in ENTR1 depleted cells, Fas localised predominantly to the limiting membranes of enlarged endosomes, where it appeared to localise to specific subdomains, however we also observed some enlarged endosomes with luminal Fas (Fig. 5a). In order to quantify the distribution of Fas across the enlarged endosomes, a line scale analysis was performed on confocal cross-sections as described before^36^. A representative line scan analysis of a confocal cross-section is shown in Fig. 5b, demonstrating the reduced luminal Fas fluorescence in ENTR1 depleted cells compared to control siRNA transfected cells. The effect was further quantified by analysing the middle fluorescence of more than 100 individual enlarged endosomes from three independent experiments (Fig. 5c). In contrast we did not see any effect on the translocation of the EGF-receptor into the lumen of MVBs, or the transferrin receptor, which is mainly recycled to the plasma membrane (Supplementary Fig 7a–d). This is also in line with our findings that EGF-receptor degradation kinetics is unchanged in ENTR1 KO cells (Supplementary Fig. 7e–g). In a second approach, we also analysed potential defects in intraluminal sorting of Fas upon depletion of ENTR1 with ‘super-resolution’ structured illumination microscopy (SIM) combined with three dimensional modelling (3D). We repeated the experiment described above but analyzed Fas localisation in enlarged vesicles using SIM instead of confocal imaging. Instead of seeing a diffuse distribution of Fas within the lumen of the enlarged endosomes, SIM allowed us to resolve a distinct luminal punctate localisation of Fas, likely representing luminal vesicles. This is further corroborated by 3D modelling of the enlarged vesicles, where Fas appears to localise to spherical stuctures within the lumen under control conditions whereas in ENTR1 depeted cells it is absent from the lumen but appeared in the limiting endosomal membrane instead (Fig. 5d, e). In summary these results suggest that the delayed degradation of Fas is due to an impaired transfer of Fas from the endosomal limiting membrane to intraluminal vesicles.

### Enhanced recycling increases Fas cell surface expression

**Fig. 6:**
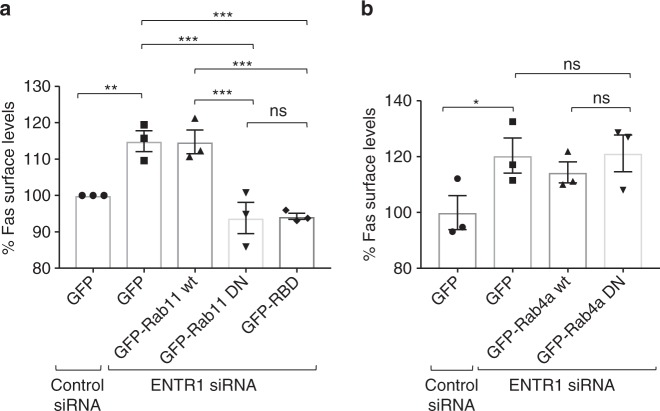
ENTR1 depletion increases Fas recycling. **a** Flow cytometry quantification of Fas surface levels after transfection of GFP-Rab11 wt, GFP-Rab11 DN (dominant negative) or GFP-RBD (Rab11 binding domain) constructs into ENTR1 siRNA depleted HeLa cells. Data was collected from three independent experiments (*n* = 3), one-way ANOVA. **b** Flow cytometry quantification of Fas surface levels after transfection GFP-Rab4a wt or GFP-Rab4a S22N DN constructs. Bar graphs represent percentage of the Alexa Fluor 633 nm median intensities. Data was collected from three independent experiments (*n* = 3), One-way ANOVA was performed, **p < 0.01, ***p < 0.001, error bars represent ± SEM

It has previously been observed that cargo accumulation at the level of early endosomes can lead to enhanced recycling^37–39^. We tested two main recycling pathways depending either on the small GTPases Rab11 or Rab4^40,41^. Using dominant negative Rab11 or the Rab11 binding domain of the Rab coupling protein (RCP) to block Rab11-dependent recycling^42^ we observed that increased Fas cell surface expression in ENTR1 depeleted cells can be completely rescued (Fig. 6a). In contrast blocking the Rab4 dependent recycling route did not affect Fas cell surface expression (Fig. 6b). We conclude that the observed increased Fas surface espression levels are due to enhanced Rab11-dependent recycling.

### ENTR1 is connected to the endolysosomal sorting machinery

**Fig. 7:**
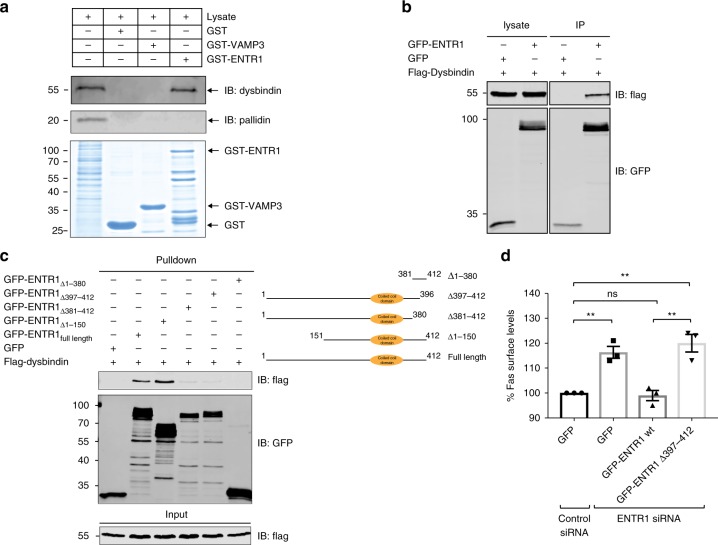
ENTR1 associates with Dysbindin to regulate Fas surface levels. **a** GST pull down of endogenous Dysbindin in HeLa cells. **b** Overexpressed Dysbindin interacts with ENTR1. GFP-Trap pulldown of flag-Dysbindin and N-terminal GFP-tagged ENTR1. HEK293 cells were transiently transfected with expression constructs for flag-Dysbindin and the indicated GFP proteins. Cells were lysed and subsequently subjected to GFP-Trap®_A pulldown. Bound proteins were detected via western blotting with anti-flag and anti-GFP antibody. **c** Binding analysis of ENTR1 with flag-Dysbindin. HEK cells were transiently transfected with the indicated ENTR1 wild-type or deletion expression constructs. After 48 h cell lysates were subjected to GFP-Trap®_A pulldown. Bound proteins were detected via western blotting with anti-flag and anti-GFP antibody. **d** Flow cytometry analysis of Fas surface levels upon ENTR1 knock-down and transfection with GFP-ENTR1 wild-type or a truncated version delta 397–412. Bar graphs represent the mean of the Alexa Fluor 633 nm median intensities. Data was collected from three independent experiments (*n* = 3), One-way ANOVA was performed, **p < 0.01, error bars represent ± SEM

**Fig. 8:**
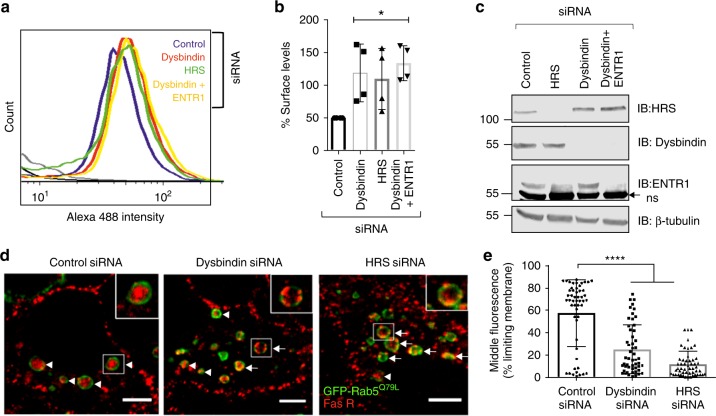
Impaired Fas sorting into intraluminal vesicles upon knock down of Dysbindin or HRS. **a** Flow cytometry analysis of Fas receptor surface levels in HeLa cells upon treatment with control (blue), Dysbindin (red), HRS (green) and Dysbindin + ENTR1 no. 3 siRNA (yellow). Shift of the peaks towards the right in all the samples as compared to the control indicates increased Alexa 488 intensity. Data was obtained from BD FACSCalibure™ flow cytometer. **b** Quantification of the flow cytometry analysis of Fas receptor levels expressed as a percentage where control is 100%. One way-ANOVA, *n* = 3,*p < 0.05, *p* = 0.0254, error bars represent ± s.e.m. **c** Immunoblot analysis of the knock-down efficiency of the indicated proteins. ns = non-specific band. ß-tubulin was used as a loading control. **d** Depletion of Dysbindin and HRS affected sorting of Fas receptors from endosomal limiting membrane to the intraluminal vesicle as compared to the control siRNA. Representative enlarged endosomes are shown in the expanded insets for each sample. Arrows indicate endosomes exhibiting limiting membrane Fas, arrowheads indicate endosomes exhibiting intraluminal Fas. Scale bar represents 5 µm. **e** Quantification of the mean fluorescence intensity found in the middle region (40–60%) of the enlarged endosomes. One way ANOVA, cells > 50 were analysed from three independent experiments (*n* = 3), ****p < 0.0001, error bars represent ± SEM

Recently, two independent proteomic studies have suggested that ENTR1 forms a complex with the protein Dysbindin^43,44^. Dysbindin interacts directly with hepatocyte growth factor regualted tyrosine kinase substrate (HRS or ESCRT-0)^45,46^, a component of the ESCRT-complex, which is mainly responsible for sorting cargo from the endosomal limiting membrane into intraluminal vesicles. Furthermore, Dysbindin together with HRS regulates the sorting of several G-protein coupled receptors to lysosomes and into intraluminal vesicles, respectively^36,45^. Thus, the interaction of ENTR1 and Dysbindin could provide a potential molecular link between ENTR1 and the endosomal sorting machinery. To test whether ENTR1 interacts with Dysbindin we applied a GST pull down assay and confirmed in line with previous proteomic results that Dysbindin interacts with a fusion protein of GST and ENTR1 but not with GST alone or GST-VAMP3, another coiled coil containing protein (Fig. 7a). Dysbindin has also been described as an obligatory component of BLOC-1 (biogenesis of lysosome-related organelles-1 complex)^47,48^. Interestingly, we did not find any Pallidin, another obligatory component of BLOC-1, in the pull-downs, suggesting that ENTR1 does not bind to the canonical BLOC-1 complex. Furthermore, we could also demonstrate interaction of ENTR1 and Dysbindin using co-immunoprecipitation (Fig. 7b). To add further evidence that binding of ENTR1 to Dysbindin is important for Fas sorting, we performed rescue experiments using a mutant version of ENTR1 with significantly reduced binding to Dysbindin. In binding site mapping experiments we could demonstrate that an ENTR1 version lacking the last c-terminal 16 amino acids almost completely abolishes interaction with Dysbindin (Fig. 7c). We could not observe any rescue expressing the mutant version of ENTR1 with reduced Dysbindin binding whereas wild-type ENTR1 could rescue Fas cell surface levels (Fig. 7d). Next we tested whether depletion of Dysbindin displays a similar phenotype with respect to Fas localisation and trafficking as observed after depletion of ENTR1. Using fluorescence flow cytometry we observed also increased Fas surface levels after Dysbindin depletion (Fig. 8a–c). This increase was not further elevated upon simultaneous knock-down of Dysbindin and ENTR1 as expected if both proteins act mechanistically together (Fig. 8b). Interestingly, we also observed increased Fas surface levels after HRS depletion (Fig. 8a, b). Altogether, this opens the possibility that Dysbindin and HRS in concert with ENTR1 regulate sorting of Fas into the lumen of multivesicular bodies. To test this hypothesis we repeated the experiments described above, analysing translocation of Fas from the limiting endosomal membrane into intraluminal vesicles after depletion of Dysbindin or after depletion of HRS, respectively. In line with our hypothesis we observed in both cases impaired translocation of Fas into the lumen of MVBs (Fig. 8d, e), however we see also some endosomes with luminal Fas. Thus, our data support the idea that ENTR1 regulates Fas sorting into intraluminal vesicles and to lysosomes via connecting to Dysbindin and ESCRT-0.

### Depletion of ENTR1 enhances Fas induced apoptosis

**Fig. 9:**
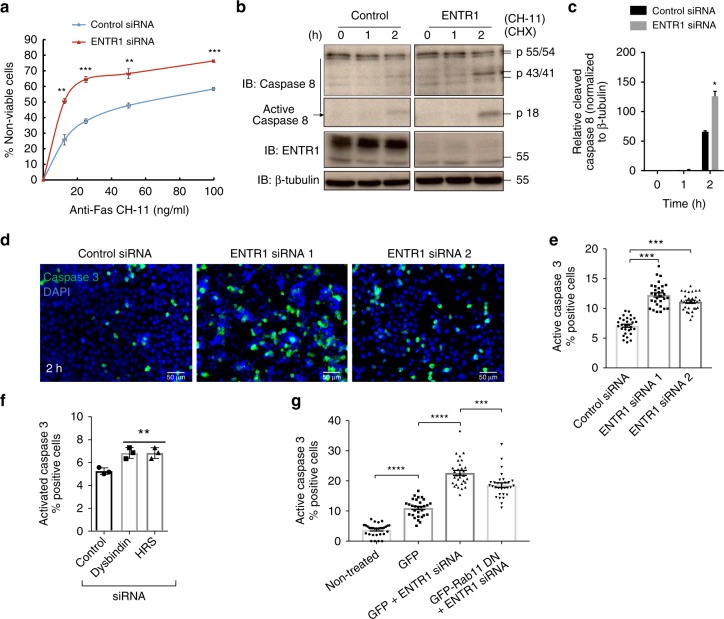
Reduced ENTR1 expression increases sensitivity to Fas induced apoptosis. **a** Cell viability was measured using MTT assay for HeLa cells treated with control or ENTR1 siRNA. A dose-dependent curve was generated indicating percentage of non-viable cells (three independent experiments, *n* = 3, **p < 0.01, ***p < 0.001, error bars represent ± s.e.m, student *t*-test. **b** Immunoblot analysis of caspase 8 cleavage upon activation of HeLa cells treated with control or ENTR1 no.3 siRNA. Cells were incubated with 500 ng/ml of anti-Fas (CH-11) antibody and 100 µg/ml of cycloheximide for the indicated time points. ß-tubulin was used as negative control. Uncropped blots are shown in Supplementary Fig. 11. **c** Quantification of cleaved caspase 8 fragments (p18) in control and ENTR1 knock-down samples normalised to ß-tubulin. X-ray films were scanned and quantified with ImageJ. Data was collected from three independent experiments (*n* = 3), ANOVA test was performed, *p < 0.05, error bars represent ± s.e.m **d** Immunofluorescence analysis of activated caspase 3 in HeLa in control or ENTR1 no.1 and no.2 siRNA cells after 2 h of sFasL (0.5 ug/ml) and CHX (50 ug/ml) treatment. **e** Quantification of the cells positive for activated caspase 3 in each condition. Data was collected from three independent experiments (*n* = 3), One-way ANOVA was performed, **p < 0.01, error bars represent ± SEM **f** Graphical representation of activated caspase 3 positive cells in control and Dysbindin or HRS siRNA treated cells. Data was collected from three independent experiments (*n* = 3), One-way ANOVA was performed, **p < 0.01, error bars represent ± s.e.m. **g** Quantification of the activated caspase 3 in untreated or treated (CH11 500 ng/ml and CHX 150 ug/ml) HeLa cells transfected with the indicated expression constructs. Data was collected from three independent experiments (*n* = 3), One-way ANOVA was performed, **p < 0.01, ***p < 0.001, error bars represent ± SEM

As described above, knock-down of ENTR1 elevates cell surface levels of Fas. To test whether this increase also correlates with enhanced Fas-mediated apoptosis we incubated HeLa cells, transfected with siRNA targeting ENTR1 or control siRNA, with CH11 and monitored the extent of apoptosis. We observed a significant increase in non-viable cells in HeLa cells treated with siRNA targeting ENTR1 compared to control siRNA treated cells. This effect was strictly dependent on CH11 concentration (Fig. 9a).

Fas is known to activate a signalling cascade which leads to proteolytic activation of procaspase 8 and 3. Thus we tested whether activation of caspase 8 and caspase 3 was also affected by ENTR1 depletion. Activation of caspase 8 can be monitored by Western-blotting via the appearance of the proteolytically cleaved active 18 kDa version of caspase 8 (in addition to a partially cleaved version of 43/41 kDa). Caspase 3 can be monitored by immunofluorescence using an antibody specifically recognising activated caspase 3. In line with increased surface expression levels of Fas and increased rate of apoptosis we observed enhanced Fas-mediated signalling as measured by activation of caspase 8 (Fig. 9b, c). We could also confirm enhanced activation of caspase 8 and elevated Fas induced apoptosis in ENTR1 depleted HCT116 cells (Supplementary Fig. 3e–h). Finally, to demonstrate that this is not only specific for CH11 as an agonistic trigger, we also demonstrated increased apoptosis rates measuring caspase 3 activation in ENTR1 depleted cells treated with a soluble version of the natural Fas-ligand (SuperFas-ligand) (Fig. 9d, e). In line with our proposed model we also detected increased apoptosis rates after knocking down of Dysbindin or HRS (Fig. 9f).

As demonstrated above we were able to rescue Fas cell surface expression to wild-type levels in an ENTR1 depleted background by blocking recycling using dominant negative Rab11 (Fig. 6a). We have repeated this experiment and measured the apoptosis rate. Surprisingly we observed only a partial rescue although we were able to rescue cell surface expression completely (Figs. 6a, 9g). This suggests that increased cell surface levels are likely not the only mechanism for the observed enhanced apoptosis.

### ENTR1 is cleaved during Fas-induced apoptosis

**Fig. 10:**
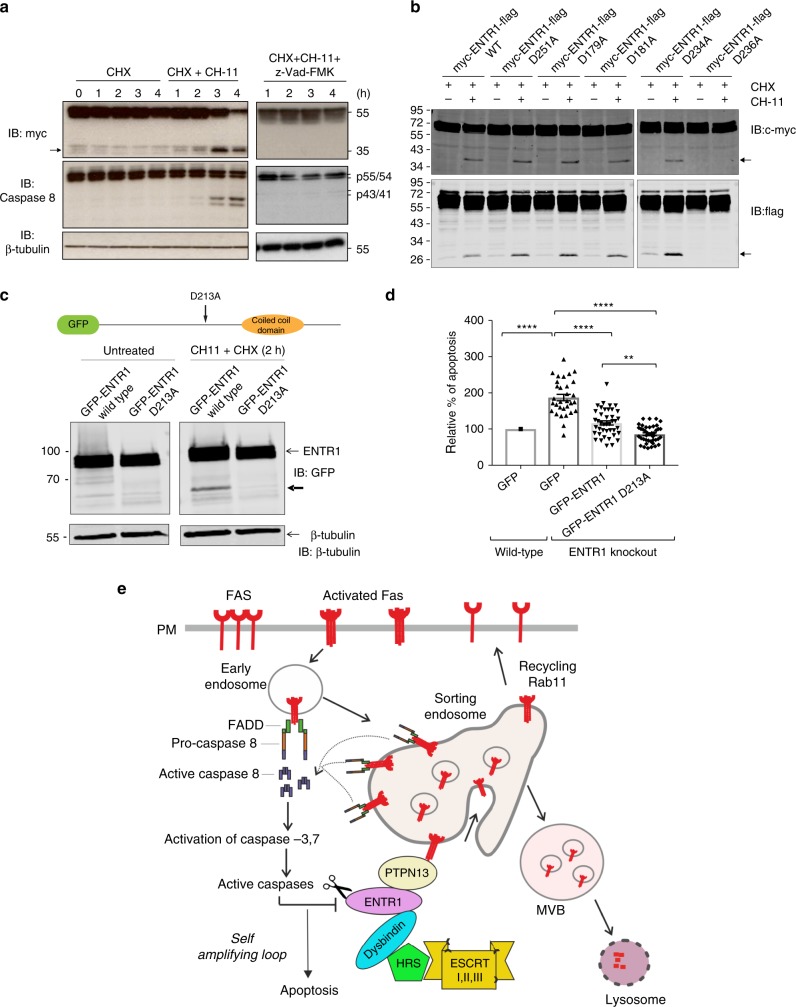
ENTR1 is cleaved during Fas induced apoptosis. **a** Overexpressed ENTR1-myc (55 kDa) was cleaved upon activation of Fas-mediated apoptotic signalling to a produce a 35–40 kDa band after 3 h of treatment (indicated by an arrow) with anti-Fas (CH-11) and cycloheximide. Cleavage of Caspase 8 (p18) was also observed only upon activation with anti-Fas (CH-11). Myc-ENTR1 was not cleaved upon activation by cycloheximide only or **b** in the presence of pan-caspase inhibitor (z-vad-FMK). **b** ENTR1 is cleaved by caspase at position aa236 (isoform1) into two fragments (indicated by arrows). Myc- and flag-tagged ENTR1 mutants D179A, D181A, D234A and D236A were created by site-directed mutagenesis using D251A mutant as a template. **c** Immunoblot showing equal protein expression of the ENTR1 rescue constructs (includes a cartoon of the ENTR1 caspase resistant construct). Cleaved ENTR1 product indicated with an arrow. beta-tubulin was used as a loading control. **d** A caspase resistant ENTR1 has increased anti-apoptotic activity compared to wild-type ENTR1. Quantification of the immunofluorescence analysis of activated caspase 3 in CH11 treated wild-type HeLa cells or ENTR1 knock-out cells transfected as indicated in the graph. Relative percentage of apoptosis is shown and HeLa wild-type GFP-transfected cells is considered as 100%. Only GFP positive cells were included in the quantification. Data was collected from three independent experiments (*n* = 3), One-way ANOVA was performed, **p < 0.01, ***p < 0.001, error bars represent ± SEM. **e** Working model for endolysosomal sorting of Fas via the ENTR1/PTPN13 complex and Dysbindin. Impaired sorting of Fas into intraluminal vesicles leads to increased recycling and elevated Fas cell surface expression. Caspase dependent cleavage of ENTR1 contributes to a positive feedback apoptotic signalling loop (PM = plasma membrane)

A recent report suggested that ENTR1 is a substrate for caspase 6^49^, an effector protease, which can be activated downstream of caspase 8 activation^50^. This finding opened the possibility that ENTR1 could be cleaved during Fas-mediated apoptosis as caspase 8 and caspase 6 are activated after Fas activation^51^. To facilitate detection of potential Fas cleavage products of ENTR1 we transfected HeLa cells with a Myc-tagged ENTR1 expression construct and monitored its expression levels after induction of apoptosis. We observed that the band of full-length Myc-ENTR1 became weaker over time; in parallel we noticed the appearance of a shorter band at around 35 kDa. This band did not appear upon adding CH11 together with the pan-caspase inhibitor z-Vad-FMK suggesting that ENTR1 is cleaved in a caspase-dependent manner (Fig. 10a). We also observed a cleavage product of similar size for endogenous ENTR1 after stimulation with CH11 (Supplementary Fig. 9a). We performed a similar experiment in the presence of more specific caspase inhibitors and observed significant reduction of cleavage upon addition of inhibitors specific for caspase 6 or caspase 8. In contrast we could not see any inhibitory effect with inhibitors for caspase 1 or caspase 3 (Supplementary Fig. 9b), suggesting that caspase 6 and 8 contribute to the cleavage of ENTR1 either directly or indirectly.

### Cleavage–resistant ENTR1 and its anti-apoptotic activity

Our finding that a negative regulator of Fas cell surface levels like ENTR1 is targeted by Fas apoptotic signalling has major mechanistic implications as cleavage of ENTR1 could enhance Fas apoptotic signalling similar as described above for the knock-down of ENTR1. To investigate this further we mapped the caspase cleavage site in ENTR1. ENTR1 has two major splicing variants and to avoid missing any cleavage sites we decided to test the longest splicing variant in our cleavage assays. We tagged ENTR1 N-terminal with a Myc-tag and C-terminal with a Flag-tag. Upon induction of apoptosis we could observe two cleavage products, one was recognised by anti-myc antibody (at around 40 kDa) and one was recognised with anti-Flag antibody (at around 27 kDa) (Fig. 10b and supplementary Fig. 9b). The molecular weight of both fragments together is roughly equal to the 65 kDa, which is what we observed for the double-tagged ENTR1 protein suggesting that there is only a single caspase cleavage site. Taking into account the molecular weights of the cleavage fragments we identified potential cleavage sites based on previously identified caspase cleavage sites in other proteins^52^. In each of these sites we mutated the conserved aspartate to alanine; in total we tested five different mutants in our apoptosis assay and identified a single cleavage site at position amino acid 236 (Fig. 10b). LC-MS/MS analysis of a tryptic digest of the 27 kDa band from immunoprecipitations of Myc-ENTR1-Flag confirmed cleavage at aspartate 236 by identification of a semi-tryptic peptide C-terminal to the cleavage site (supplementary Fig. 9c, d). All rescue experiments above have been performed with splicing variant 2, which is the 13 amino acid shorter variant of ENTR1. To be consistent we introduced the same point mutation into splicing variant 2 (D213A) and confirmed that this leads to caspase-resistance (Fig. 10c). Next, we tested the ability of this mutant to rescue enhanced apoptosis in an ENTR1 knock-out background. We generated HeLa cells lacking ENTR1 using CRISPR/Cas9, which demonstrated as expected increased Fas cell surface levels and enhanced sensitivity to Fas induced apoptosis (Fig. 10d and supplementary Fig. 8d). We rescued enhanced apoptosis by transfecting either wild-type ENTR1 or caspase-resistant D213A ENTR1. As expected wild-type ENTR1 was able to rescue the enhanced apoptosis, however caspase-resistant ENTR1 was significantly more effective in rescuing apoptosis compared to wild-type ENTR1 (Fig. 10d). This result suggests that blocking caspase-induced cleavage of ENTR1 reduces Fas apoptotic signalling.

### ENTR1/PTPN13/Fas expression levels in colon cancer tissue

We have shown previously that ENTR1 expression is upregulated in colon cancer tissue^26^. This prompted us to investigate expression levels of ENTR1, PTPN13 and Fas in colon cancer samples. We confirmed our previous finding that ENTR1 is upregulated in colon cancer tumours compared to non-tumorous tissue, similarly we also observed elevated PTPN13 expression in colon cancer tissue samples, whereas Fas expression was downregulated (Supplementary Fig. 10a–d). Interestingly, when comparing the expression of ENTR1/PTPN13 with Fas a significant proportion of colon cancer samples exhibit upregulated ENTR1 or PTPN13 and concomitantly downregulation of Fas expression levels. 78% of the colon cancer samples with elevated ENTR1 and 73% of the colon cancer samples with elevated PTPN13 showed downregulation of Fas expression (Supplementary Fig. 10e and f). This is in line with our findings that ENTR1 and PTPN13 are important for sorting Fas for lysosomal degradation.

## Discussion

In this study we identified the endosome associated trafficking regulator 1 (ENTR1) as a negative regulator of Fas cell surface levels and Fas-induced apoptosis. We provide evidence that ENTR1 is an important player of Fas endocytic trafficking regulating Fas degradation by contributing to the sorting of Fas into intraluminal vesicles of multivesicular bodies and thereby shutting down Fas-dependent signal transduction. Furthermore, we show that ENTR1 is cleaved during Fas-induced apoptosis revealing a positive feedback loop of apoptotic signalling.

A key mechanism of cells to regulate responsiveness to particular ligands is by regulating cell surface levels of their corresponding plasma membrane receptors and altered receptor recycling can be part of disease mechanism^53,54^. We show here that depletion of ENTR1 correlates with enhanced sensitivity to Fas induced apoptosis. This is of particular importance, because several cancer cells express low surface levels of Fas and thereby escaping potential Fas induced apoptosis, however the underlying molecular mechanism is often unclear^16,17,19,20,22,55^. Here we present evidence for a role of ENTR1 and the multi-PDZ domain protein PTPN13 in post-endocytic sorting of Fas to lysosomes. We could not detect any effect of ENTR1 depletion at the step of internalisation of Fas, which is in line with our previous finding that ENTR1 localises to early and recycling endosomes^26^. However, we observed significant impairment in the transition of Fas from the early endosomal compartment to lysosomes. Similar observations have been made before if cargo cannot enter intraluminal vesicles of MVBs. In such cases cargo remains in the limiting endosomal membrane and cannot enter the lysosomal compartment^36,45,56^. The main machinery regulating sorting of cargo and formation of intraluminal vesicles are the ESCRT complexes (0–IV)^57,58^. Several recent studies show a role for Dysbindin in cargo delivery to lysosomes. Dysbindin forms a complex with HRS and is important for post-endocytic sorting of the G-protein coupled D2 dopamine receptor^45^. Furthermore, Dysbindin forms also a complex with the G-protein Gα_s_ and the G-protein coupled receptor associated binding protein-1 (GASP1) regulating the post-endocytic trafficking of a variety of receptors including the chemokine receptor type 4 (CXCR4)^36^. Moreover, it was shown that Dysbindin is important for the entry of CXCR4 into intraluminal vesicles^36^. Finally, depletion of Dysbindin correlated with increased cell surface levels of CXCR4 and delayed degradation kinetics, very similar to what we have observed for Fas after ENTR1 depletion. We show here that ENTR1 interacts with Dysbindin and that this interaction is important to regulate Fas cell surface levels. We also show that depletion of Dysbindin leads to impaired translocation of Fas into intraluminal vesicles of multivesicular bodies, increased Fas cell surface levels and enhanced sensitivity to Fas induced apoptosis. Taken together, this strongly suggests that ENTR1 regulates sorting of Fas into intraluminal vesicles via the Dysbindin-HRS axis. Our finding that depletion of HRS also increased Fas cell surface levels and impaired endo-lysosomal sorting of Fas further supports this. Dysbindin is a well-established component of BLOC-1, which is implicated in intracellular protein trafficking and in the biogenesis of specialised organelles of the endo-lysosomal system^59,60^. Our data point however to a second role of Dysbindin likely independent of BLOC-1 in regulating cargo transport into intraluminal vesicles. In line with this is our observation that we could not find Pallidin, which is another obligatory BLOC-1 component, in ENTR1 pulldowns. Dysbindin found in complex with ENTR1 represents likely a minor fraction of total Dysbindin as most Dysbindin appears to cofractionate with BLOC-1 in cells.

In summary, we propose a model where ENTR1 binds to Fas via PTPN13 and connects to the ESCRT machinery via Dysbindin regulating post-endocytic sorting of Fas. In this model, increased Fas cell surface expression is explained by the accumulation of Fas at early endosomes and increased Rab11-dependent recycling (Fig. 10e). Interestingly, increased recycling has also been demonstrated in Dysbindin knock-out cells for the dopamine D2 receptor^45^.

The endosomal compartment can serve as a major site for Fas-mediated DISC formation and caspase-8 activation and Fas internalisation is a major requirement for Fas-mediated apoptosis in type I cells, cells which rely mainly on the extrinsic apoptosis pathway without major involvement of the mitochondrial-dependent apoptosis pathway^4–6,50^. The longer retention time of Fas in the endosomal compartment observed after ENTR1 depletion is likely to contribute to enhanced apoptotic signalling in ENTR1 depleted cells.

A number of proteins involved in intracellular trafficking have been shown to be substrates for caspases^52^. Here we show that ENTR1 is a target for caspase-mediated cleavage after Fas activation. Together with our ENTR1 depletion results described above, this suggests that proteolytic decrease of endogenous ENTR1 protein impairs Fas degradation and increases apoptotic signalling which will in turn increase caspase mediated cleavage of ENTR1 and other downstream targets resulting in a positive feedback loop of apoptotic signalling (Fig. 10e). As predicted by our model we could observe enhanced anti-apoptotic activity with a caspase-resistant version of ENTR1 compared to wild-type ENTR1.

Finally, we have established here that ENTR1 and PTPN13 act together to regulate Fas cell surface levels. This gives also deeper insights into the mechanism how PTPN13 can regulate Fas sensitivity in cancer. Previously, it has been noted that there is a negative correlation between PTPN13 expression and Fas induced apoptosis in cancer cell lines^16,22,24,55,61–63^ and in colon cancer in vivo^64,65^. Furthermore, a recent study demonstrates that uncoupling binding of Fas to PTPN13 by adding a peptide derived from the PDZ binding motif of Fas restores Fas sensitivity and decreased the growth of colon cancer xenografts in a mouse cancer model^65^.

Our identification of ENTR1 as a negative regulator of Fas-mediated apoptosis has revealed insights into key aspects of the endolysosomal sorting of Fas and how this regulates sensitivity to Fas-induced apoptosis; however further investigations are necessary to fully understand the complex interplay of Fas membrane trafficking and its signal transduction.

## Methods

### Antibodies and reagents

The following antibodies were used in this study: ENTR1 (15969­1­AP, 1:300 for IF) Proteintech, Manchester, UK; ENTR1 (HPA029303, 1:250 for IB), ß-tubulin (T4062, 1:5000 for IB), Anti­Flag M2 (1:1000 for IB) Sigma‐Aldrich, England; Fas (C‐20, 1:500 for IB), N‐Cadherin (13A9, 1:500 for IB), rabbit anti-c-Myc (A-14, 1:100 for IB), rabbit anti-Fas (C-20, 1:500 IB), rabbit anti-FAP1 (H-300, 1:500 for WB), goat anti‐EEA­1 (N­19, 1:100 for IF) Santa Cruz (USA). Anti‐Fas clone CH­11 (1:500 for IF and FC) Millipore and anti‐Fas clone DX‐2 (1:500 for FC) Biolegend (USA). CD71 (D7G9X) XP (1:2000 for IB), EGF (2232, 1:1000 for IB), rabbit anti‐EEA‐1 (C45B10, 1:300 for IF), anti‐HA (6E2, 1:1000 for IB), anti-Caspase 8 (1C12, 1:1000 for IB), anti-cleaved caspase 3 (Asp175, 1:400 for IF), anti-HRS (14346, 1:1000 for IB) Cell signalling (USA). Anti-Dysbindin (ab124967, 1:1000 for IB), mouse anti-pallidin (ab169037, 1:500 for IB), rabbit anti-Lamp‐1 (1: 800 for IF) Abcam (Cambridge, UK). Alexa 594 conjugated. Transferrin Invitrogen and anti-CD261/TRAIL­R1 FITC conjugated (clone DR­4‐02) Life technologies. Rabbit anti-PTPN13 (NB100-56139, 1:500 for IF; 1:1000 for IHC) from Novus Bio. Rabbit anti-GFP was a kind gift from Andrew Peden, University of Sheffield (1:2000 for IB). Caspase Family Inhibitor Set (K107) was acquired from BioVision Inc.; Super Fas Ligand (sFasL) from Enzo Life Sciences and GFP-Trap®_A from ChromoTek GmbH (Planegg-Martinsried, Germany).

### Cell Culture, DNA and transfection

HeLa and HCT116 cells were from Sigma-Aldrich. Cells were cultured and maintained in Dulbecco’s modified Eagle’s medium (DMEM) high glucose (Invitrogen) supplemented with L‐glutamine, 10% Fetal bovine serum (Invitrogen), 1% penicillin and streptomycin (Invitrogen) at 37 °C under 5% CO_2_. Full length‐ ENTR1 was from Origene (Rockville, MD, USA). ENTR1 constructs were subcloned into pcDNA3 vector (Invitrogen, Darmstadt, Germany) harbouring an N-terminal EGFP. Full length ENTR-1 was subcloned into a pGEX-6-P1-GST vector (GE Healthcare, München). ENTR1 P167A and E171A mutants and ENTR1 caspase cleavage mutants were generated by site-directed mutagenesis (Quick change II XL by Agilent technologies). pGEX h-VAMP3, Rab5 (Q79L)­EGFP and RBD-GFP^66^ constructs were a kind gift of Dr Andrew Peden. Rab11 wt-GFP, Rab4a wt-GFP and Rab4a S22N-GFP constructs were a kind gift from Prof Elizabeth Smythe. HA-Fas and E-­cadherin from Erdmann lab. pCS2 HRS‐RFP was a gift from Edward De Robertis (Addgene plasmid #29685). pCMV3‐C‐Flag human Dysbindin gene was from Sino Biological Inc. (HG15072­CF). Cells were transfected with DNA using Lipofectamine 2000 (Invitrogen) according to the manufacturer’s protocol.

### RNA interference and rescue experiments

All­star negative control siRNA, Hs_ENTR1_6 (CGACGCACUGAAAGAUGAA), which is siRNA1 and Hs_ENTR1_7 (ACUGAAUCUUGUUGCCGAA) which is siRNA 2 were from Qiagen. Pre‐designed ENTR1­s21238 (CCACGUCGUGAAACUAAAA), which is siRNA 3 was from Invitrogen. SiRNA for Dysbindin (CAGCAAAUCUGACUCAUUU) was from Ambion, Life technologies^45^ and for Hrs (CGACAAGAACCCACACGUC) from Dharmacon^37^. PTPN13 siRNA1 (AAGUAAGCCUAGCUGAUCCUGUU), PTPN13 siRNA2 (CAGAUCAGCUUUCCUGUAAUU) were both from Dharmacon. For the rescue experiments, ENTR1 constructs were made siRNA resistant by mutating three consecutive nucleotides with site directed mutagenesis kit (Quick change II XL by Agilent technologies) according to the manufacturers’ protocol.

### Flow cytometry

Cells were detached from the culture dish with trypsin‐EDTA for 5 min and washed once with 1% BSA/PBS and incubated with either Alexa conjugated or unconjugated primary antibodies for 1 h at 4 °C. After labelling with secondary antibody, cells were analysed immediately by BD LSRII™ or BD FACSCalibur™ flow cytometers (BD Biosciences). Secondary antibody alone was used as the negative control for determining specificity of the signal.

### Immunofluorescence analysis

Images were acquired using the Perkin Elmer Ultraview VoX spinning disc confocal microscope and Olympus epifluorescence microscope. Super­resolution microscopy was performed using Deltavision/GE OMX optical microscope for structured illumination (3D‐SIM). Co‐localisation analysis was performed on confocal sections showing maximum intensity for Lamp‐1 or EEA‐1 and Fas antigens using JACoP plugin in Image J software. Threshold levels were adjusted uniformly across the conditions to reduce background noise from the analysis. Occurrence of co‐localisation was calculated using Pearson’s correlation coefficient (PCC) and quantification of the co­localisation was obtained using overlap coefficients (k1 and k2). Endosomal lumen localisation was analysed following established protocols [34]. A straight line was drawn across the diameter of each enlarged endosome. Intensity was normalised to account for varying endosome sizes. Luminal membrane was visualised via Rab5 Q79L-GFP, Fas was visualised via anti-Fas antibody. The first and second maximum peaks of the GFP fluorescence were marked as 0 and 100 respectively representing the luminal membrane boundaries. Middle fluorescence values (40–60) for Fas were calculated from raw pixel intensities obtained from line scale analysis using Image J.

### Protein interaction experiments and immunoblotting

For GFP-Trap pulldown, cells were lysed in lysis buffer (50 mM HEPES, pH 7.5, 150 mM NaCl, 1.5 mM MgCl_2_, 1 mM EDTA, 10% glycerol, 1% Triton X-100). Cell lysates were incubated for 3 h with GFP-Trap®_A at 4 °C. After three washing steps with lysis buffer, bound proteins were eluted in Laemmli buffer at 95 °C and separated by SDS–PAGE. Proteins were transferred onto nitrocellulose membrane, incubated with the relevant antibodies and detected using the Odyssey Sa Infrared Imaging System (LI-COR Biosciences, Ltd, UK). Uncropped images of all western-blots displayed in this study are provided as supplementary Fig. 11.

### GST pull-down assay

Lysates were obtained from 60 mm confluent dishes of HeLa cells and 10% of the pre-cleared protein extract was used as input representing total protein levels. The remaining cell lysate was added to 50 μl of GST fusion protein or GST control beads slurry and incubated for 4 h at 4 °C and constant mixing. After incubation, samples were spun at 6030 g for 2 min at 4 °C. Beads were washed five times with 500 μl of cold 0.5% Triton X-100 in PBS by centrifuging at 6030 g for 2 min between each of the washing steps. Finally, proteins were eluted from the beads by adding 2x Laemmli buffer followed by heating at 95 °C for 5 min. Proteins were loaded on 10% acrylamide gels and separated by SDS-PAGE.

### Generation of ENTR1 knock-out cell lines

Guide RNAs were designed with CRISPOR online tool providing the target sequence of ENTR1. For ENTR1 gRNA design exon 4 was targeted as targeting exons 1 and 2 would not affect ENTR1 isoform 3 (information obtained from UNIPROT). gRNA sequence included 4 nucleotides overhangs compatible with desired vectors and a PAM motif. gRNAs were cloned in px458-GFP vector^67^. Cells were transfected with 2 μg of px458-GFP plasmid containing gRNA for ENTR1. After 48 h, cells were trypsinised, re-suspended in 1 mL of DMEM supplemented with 10% FBS and centrifuged at 94 g for 5 min. Supernatant was removed and cells were re-suspended in 500 μl of Opti-MEM. Cell suspension was transferred to flow cytometry tubes and kept on ice. Sorting of GFP positive single cells into 96-well plates was performed with BD FASC Aria Ilu (Flow cytometry Core Facility, The University of Sheffield). Sorted single cells were incubated in 96-well plates and screened for colonies after 2–3 weeks. Colonies generated from single cells were sequentially expanded. At this point, genomic DNA was extracted and region of interest was amplified using ENTR1 specific primers (forward primer GCCACACTCATGCACGATTC, reverse primer TCAGCCACCTTCACACTTCC). Western blot analysis was then used to screen for positive knock-out clones. Positive ENTR1 knock-out clones were subsequently expanded into 10 cm dishes and used for further experiments.

### Apoptosis assay monitored by cleaved Caspase 3

HeLa cells were seeded on coverslips in 12-well plates at the appropriate cell density and 24 h later transfected with either control or ENTR1/Dysbindin/HRS siRNA. Upon 72-h incubation cells were treated with CH11 (500 ng/mL) or SuperFas soluble ligand (0.5 μg/mL) and cycloheximide (50 μg/mL) in DMEM medium supplemented with 10% FBS for 2 h. Cells were subsequently fixed with 4% PFA and standard immunofluorescence protocol was performed using anti-cleaved Caspase 3 (Asp175, Cell Signalling). Finally, coverslips were imaged in Motorised Olympus BX61 Epifluorescence microscope by taking ten random pictures of 20x magnification from every condition. Quantification was based on the percentage of positive cells for cleaved Caspase 3 (representing apoptotic cells) with respect to the total number of cells.

### Cell surface biotinylation

Cells were cultured on 60 mm dishes incubated with EZ‐Link Sulfo‐NHS‐SS­Biotin (Thermo Fisher Scientific) at a concentration 0.25 mg/ml in ice cold PBS. Cells were washed with 1% BSA/PBS to quench any unbound biotin and lysed with 1% Triton X‐100 in PBS supplemented with protease cocktail inhibitors (Roche) to pull down biotinylated proteins with NeutrAvidin agarose beads (Thermo Fisher scientific).

### Endocytosis assay

HeLa cells were treated with control or ENTR1 siRNAs for 72 h. Cells were harvested using trypsin-EDTA. Only surface population of Fas receptors was labelled using 1 μg/ml of Anti-Fas CH-11 antibody in ice-cold 1% BSA/PBS solution for 1 h at 4 °C with constant rotation. Unbound primary antibody was removed by washing the cells three times with 1% cold BSA/PBS at 166xg for 2 min. Washed cells were resuspended in pre-warmed DMEM media and incubated at 37 °C for various time points. At the end of each time point, endocytosis was stopped by incubating the cells on ice. Cells were labelled with Alexa 488 conjugated secondary antibody for 1 h at 4 °C with constant rotation and washed thrice to remove any unbound labelling antibody. Samples were analysed immediately using BD LSR II Flow cytometer or BD FACSCalibur™. Unstained and secondary only labelled HeLa cells were also analysed as negative controls.

### Endogenous Fas degradation assay

HCT-116 wild-type and HCT-116 ENTR1 knock-out (clone 4) cells were seeded in 6-well plates and incubated until confluence was reached. Cells were treated with Fas CH11 agonistic antibody (500 ng/mL) and cycloheximide (150 μg/mL) diluted in DMEM medium supplemented with 10% FBS at different time points ranging from 0 to 2 h. After induction of Fas-mediated apoptosis, cells were lysed with 1% Triton in 1x PBS lysis buffer plus protease cocktail inhibitor (Roche). Protein concentration was equalised in all samples. 4x Laemmli buffer was added to protein extracts and samples were boiled at 95 °C for 5 min and subsequently analysed by western blotting. Immunoblotting for β-tubulin was used as a loading control and transferrin receptor as a control for cell surface receptor, which is not affected by Fas stimulation.

### Blocking recycling

HeLa cells were co-transfected with ENTR1 siRNA and either Rab11 wt-GFP, Rab 11 DN-GFP, Rab4a wt-GFP, Rab4a DN-GFP or RBD-GFP (Rab-binding domain of the Rab coupling protein). 72 h post-transfection, cells were labelled as described above and Fas cell surface expression was measured with the LSRII flow cytometer.

### Mapping of caspase cleavage site and caspase inhibitor assay

ENTR1 protein sequence was manually screened for potential cleavage sites resembling other known caspase cleavage sites^52^. In total five potential sites were further investigated and the conserved aspartate in each oft he sites was mutated to alanine. HeLa cells were transfected with double-tagged wild-type ENTR1 or with individual cleavage site mutant constructs and twenty-four hours post-transfection, cells were treated with 200 ng/ml CH11 and 2.5 µg/ml cycloheximide for 3 h at 37 °C, then lysed in NETN-buffer (100 mM NaCl, 20 mM Tris-HCl, pH 8.0, 0.5 mM EDTA, 0.5% NP-40). Total protein concentration was determined using DC^TM^ Protein Assay (Bio-Rad Laboratories, Inc.), protein samples were analysed by western blotting to assess cleavage of ENTR1. For the caspase inhibitor assays HeLa cells were transfected with a double-tagged wild-type ENTR1 expression construct. Twenty hours post-transfection, cells were pre-treated with 5.0 µM of the corresponding caspase specific inhibitors for 1 h at 37 °C and subsequently with 200 ng/ml CH11 and 2.5 µg/ml cycloheximide for 3 h at 37 °C. Further procedure was as described for mapping of the caspase cleavage site.

For mass spectrometry analysis HeLa cells were transfected with Myc-ENTR1-Flag expression construct. 48 h after transfection cells were stimulated with anti-CH11 for 3 h, cells were lysed and lysates were subjected to anti-Flag immunoprecipitation using anti-Flag agarose. Bound proteins were washed and eluted with Flag peptide. Eluted proteins were separated by SDS-gel electrophoresis and stained with Coomassie Blue. The 27 kDa band was excised and in-gel digestion was performed as reported previously^68^. Extracted peptides were analysed by nanoflow LC-MS/MS using an Orbitrap Elite (Thermo Fisher) hybrid mass spectrometer equipped with a nanospray source, coupled to an Ultimate RSLCnano LC System (Dionex). The system was controlled by Xcalibur 2.1 (Thermo Fisher) and DCMSLink 2.08 (Dionex). Peptides were desalted on-line using a micro-Precolumn cartridge (C18 Pepmap 100, LC Packings) and then separated using a 40 min RP gradient (4–32% acetonitrile/0.1% formic acid) on an EASY-Spray column, 15 cm × 50 µm ID, PepMap C18, 2 µm particles, 100 Å pore size (Thermo). The LTQ-Orbitrap Elite was operated with a cycle of one MS (in the Orbitrap) acquired at a resolution of 60,000 at m/z 400, with the top 20 most abundant multiply-charged (2+ and higher) ions in a given chromatographic window subjected to MS/MS fragmentation in the linear ion trap. An FTMS target values of 1e6 and an ion trap MSn target value of 1e4 was used and with the lock mass (445.120025) enabled. Maximum FTMS scan accumulation time of 500 ms and maximum ion trap MSn scan accumulation time of 100 ms were used. Dynamic exclusion was enabled with a repeat duration of 45 s with an exclusion list of 500 and exclusion duration of 30 s. MS data was analysed data using MaxQuant version 1.6.0.16^69^. Data was searched against a human UniProt sequence databases (downloaded June 2015) using following search parameters: trypsin/AspC in semi specific mode, 7 ppm for MS mass tolerance, 0.5 Da for MS/MS mass tolerance, with Acetyl (Protein N-term), Oxidation (M), carbamidomethyl (C) and Phospho (STY) set as variable modifications. A protein FDR of 0.01 and a peptide FDR of 0.01 were used for identification level cut offs.

### Immunohistochemistry for colon cancer tissue microarrays

Paraffin-embedded tissue arrays were acquired from US Biomax (CO242b). First, tissue array was deparaffinised by immersing slides twice in xylene for 10 min, followed by immersions in 100, 95 and 70% of ethanol 5 min each. Slides were immersed in water and rinsed twice in 1x PBS for 5 min. In order to inactivate endogenous peroxidase activity, slides were incubated for 20 min with 3% H_2_O_2_. After 5 min wash in 1x PBS, antigen retrieval was carried out by cooking slides for 10 min in the microwave, immersed in Na-Citrate buffer. Next, tissue array was stained according to Vectastain ABC Elite Universal kit (Vector labs) manufacturer’s protocol and subsequently incubated in peroxidase substrate (ImmPACT DAB chromogen) until the desired stain intensity was developed. Reaction was stopped and finally, slides were transferred into 95% ethanol (2 changes for 2 min each), 100% ethanol (2 changes for 2 min each) and xylene (3 changes for 5 min each) to dehydrate and clear slides. Excess of solutions was drained from slides and dried for 10 min. Coverslips were mounted in Vectamount (non-aqueous mounting medium) and dried for at least 24 h. The imaging and analysis of the tissue microarrays was performed at the Sheffield Institute for Translational Neuroscience (SITraN). Slide scanner NanoZoomerXR was used for the imaging and the software used to analyse the cores was Visiopharm.

### Statistical analysis

Prism (Graph Pad software) was used to analyse data obtained from multiple independent experiments. The statistical significance was tested using student t‐test between two groups or analysis of variance (ANOVA) between more than two groups. Multiple comparisons procedures were applied as and when required. Correlation statistical analysis were performed by using Pearson R test and Mander’s overlap coefficient R was used to quantify co-localisation.

### Reporting summary

Further information on research design is available in the Nature Research Reporting Summary linked to this article.

## Supplementary information

Supplementary Information

Reporting Summary

## Data Availability

The data that support the findings of this study are available from the corresponding author upon reasonable request.
