# Supplementary material for: Apoptotic signalling targets the post-endocytic sorting machinery of the death receptor Fas/CD95
Source: Nat Commun. 2019 Jul 15;10:3105. doi: 10.1038/s41467-019-11025-y (PMC6629679; doi:10.1038/s41467-019-11025-y)
Supplement: Supplementary file 1 — Supplementary Information [file 41467_2019_11025_MOESM1_ESM.pdf]

SUPPLEMENTARY FIGURES

Sharma S. at al. Apoptotic signalling targets the post-endocytic sorting machinery of the death receptor Fas/CD95

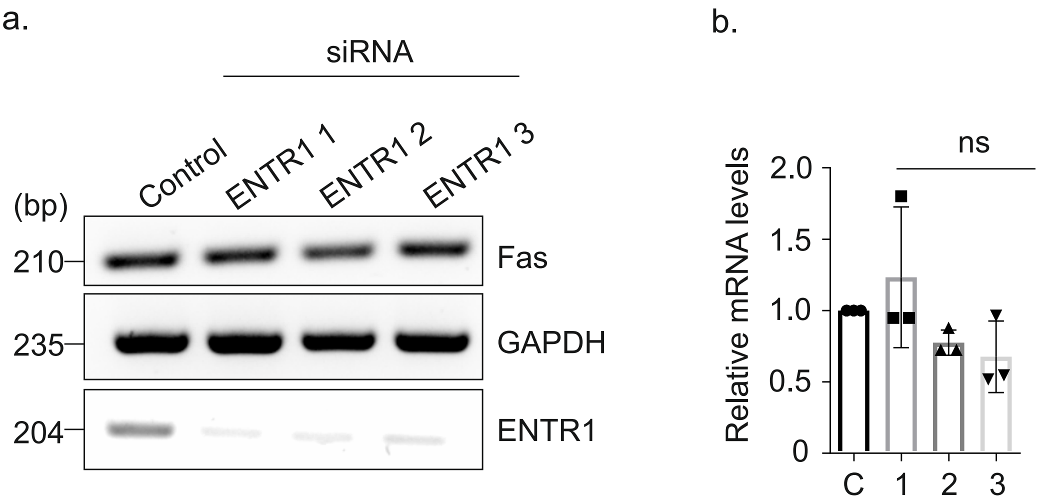

Supplementary Figure 1: Depletion of ENTR1 does not affect gene expression levels of *Fas* (a) Qualitative analysis of the changes in gene expression levels of *Fas* in HeLa cells treated with control or three different siRNA against ENTR1. Gene expression levels of *GAPDH* were used as a negative control. Depletion of ENTR1 with all three siRNA decreased the expression levels of ENTR1 effectively. (b) Graphical representation of the relative mRNA levels of *Fas* receptors in ENTR1 knock-down samples (three different siRNA against ENTR1 represented as 1, 2 and 3) as compared to the control siRNA knock-down sample shown as C. ΔΔCt, Pfaffl method was used to calculate the relative gene expression where *GAPDH* was used as the normalizer. Data was collected from three independent experiments (n=3) with internal triplicates. Ordinary One-way ANOVA was performed to test if the differences among the means were significant (p=0.1480; ns= not significant), error bars represent ±s.e.m.

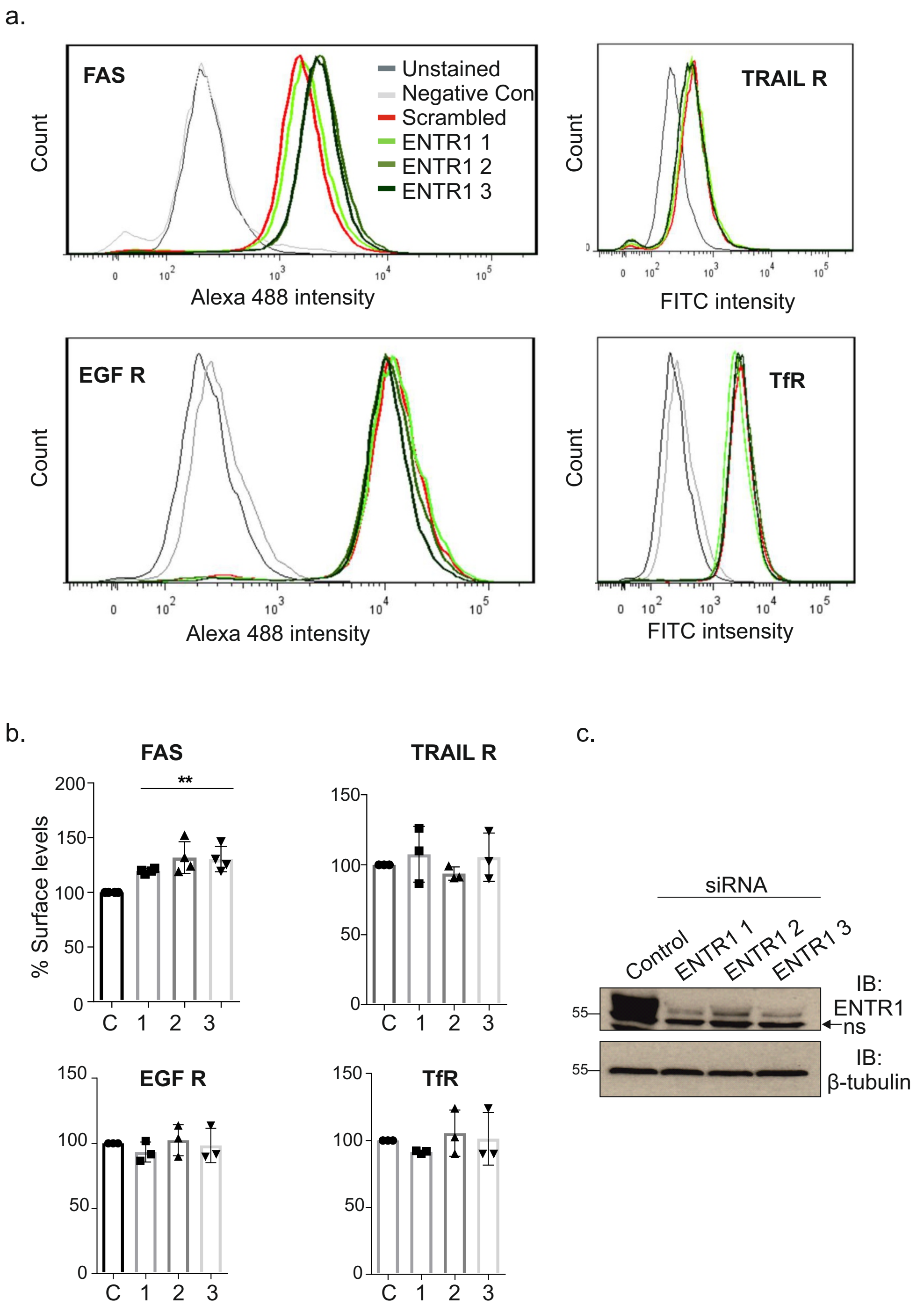

Supplementary Figure 2: Depletion of ENTR1 increases surface levels of Fas receptors in HeLa cells (a) Flow cytometry analysis of surface levels of the indicated receptors in ENTR1 (no.1, 2 and 3) and control siRNA treated HeLa cells. Live cells were stained with specific primary antibodies followed by Alexa 488 conjugated secondary antibody in some cases and observed via BD LSRII flow cytometer. (b) Bar graphs represent mean intensities from at least three independent experiments (n=3, **$p<0.05$, $p=0.0015$ for Fas, one-way ANOVA). Error bars represent ± s.e.m. (c) Immunoblot analysis of the knock-down efficiency of ENTR1 upon treatment with three different siRNA against ENTR1 and control siRNA. Arrow indicates the non-specific band (ns). ß-tubulin was used as a loading control. Uncropped blots are shown in supplementary figure 11.

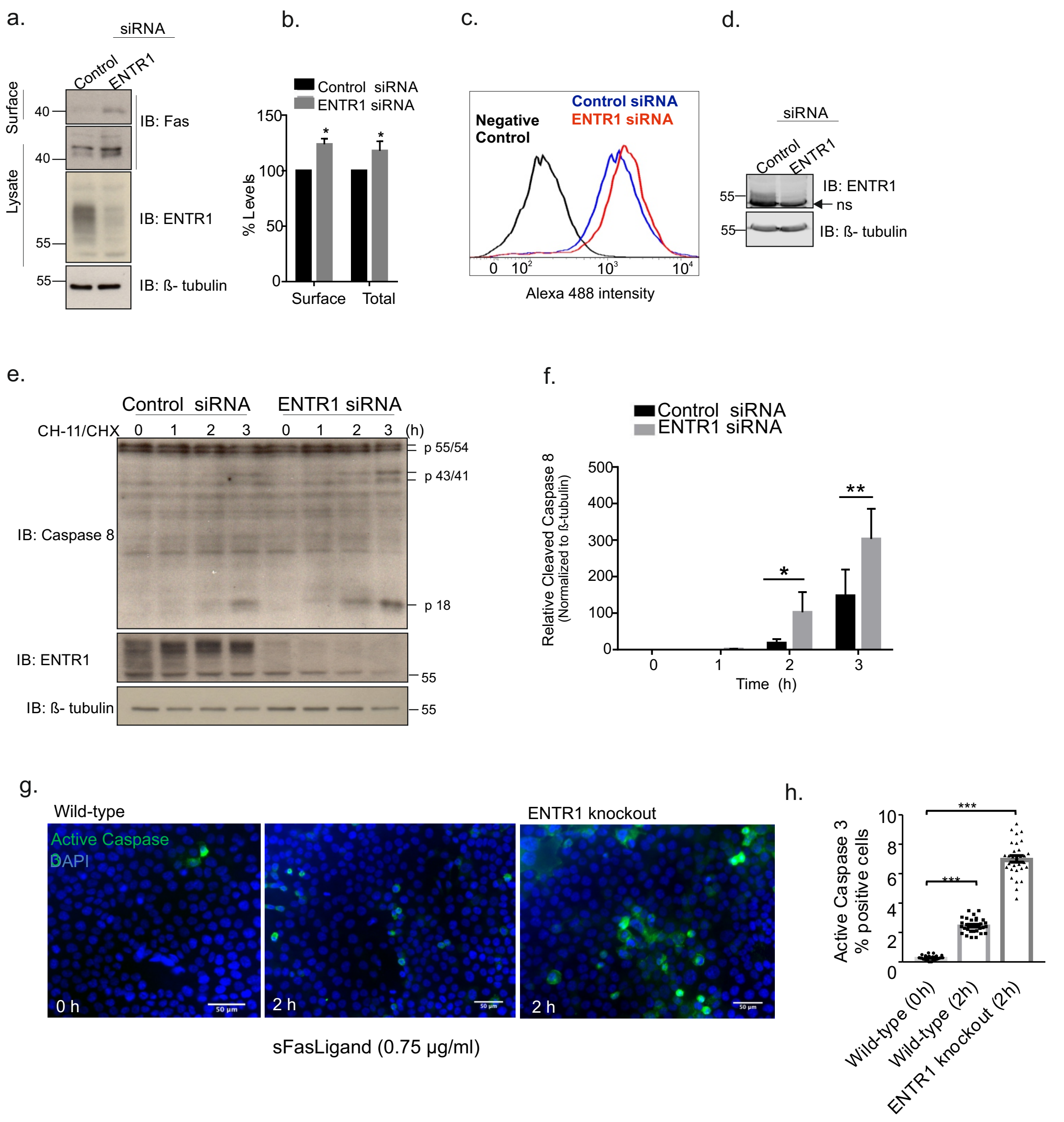

Supplementary Figure 3: Depletion of ENTR1 affects levels of Fas and apoptotic signalling in HCT116 cells (a) Immunoblot analysis of HCT116 cells treated with control or ENTR1 no.1 siRNA. The surface fraction was surface biotinylated and purified with streptavidin coated beads. Lysate was tested for total levels of ENTR1 and its knock-down efficiency and ß-tubulin acted as a loading control. Uncropped blots are shown in supplementary figure 11. (b) Quantification of immunoblots for surface or total levels of Fas receptors was performed using Image J. The levels were normalized to their respective tubulin controls and expressed as a percentage. Data was collected from three independent experiments (n=3, student's unpaired t-test, **$p<0.05$, $p=0.0100$). Error bars represent ±s.e.m (c) Flow cytometry analysis for Fas receptor levels in HCT116 cells treated with ENTR1 no.3 siRNA and control siRNA. Histograms represents intensity levels of Fas receptors in siRNA treated cells (red and blue) and negative control (black) as indicated. X-axis represents intensity of Alexa 488 conjugated antibody signal in the samples. Y-axis represents cell count. (d) Immunoblot analysis of the knock-down efficiency of ENTR1 in control or ENTR1 siRNA treated cells. ß-tubulin was used as a loading control and uncropped blots shown in supplementary figure 11. (e) Immunoblot analysis of caspase 8 cleavage upon activation of HCT116 cells treated with control or ENTR1 no.2 siRNA. Cells

were incubated with 500 ng/ml of Anti-Fas (CH-11) antibody and 50 µg/ml of cycloheximide for the indicated time points. ß-tubulin was used as negative control. (f) Quantification of cleaved caspase 8 fragments (p18) in control and ENTR1 knockdown samples normalized to ß-tubulin. X-ray films were scanned and quantified with ImageJ. Data was collected from three independent experiments (n=3), ANOVA test was performed, $*p<0.05$, error bars represent ±s.e.m. (g) Immunofluorescence analysis of activated caspase 3 in HCT116 wild-type or ENTR1 knockout (Clone 4) cells after 2 hours of treatment with sFasL (0.5 ug/ml). (h) Quantification of the cells positive for activated caspase 3 in each condition. Data was collected from three independent experiments (n=3), One-way ANOVA was performed, $**p<0.01$, error bars represent ± SEM

a.

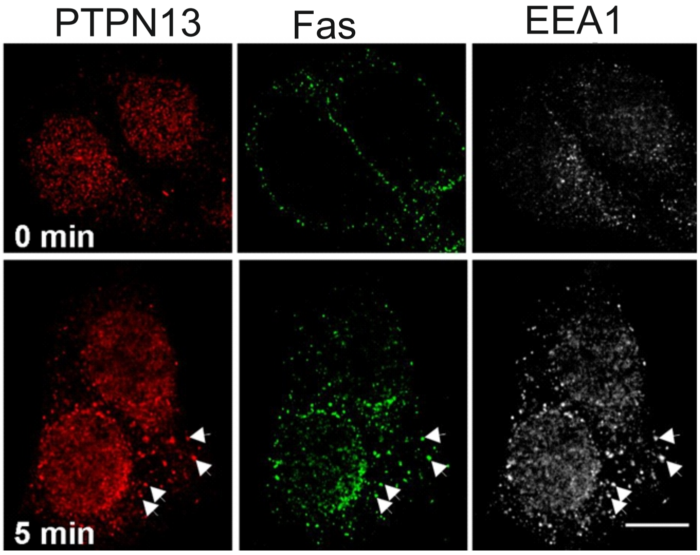

Supplementary Figure 4: Fas and PTPN13 colocalise in EEA1 positive endosomes. Immunofluorescence analysis of PTPN13 (red), FasR (green) and EEA-1 (white) in HeLa cells unstimulated or stimulated with 1µg/ml anti Fas CH-11 antibody for the indicated time points. Arrowheads highlight punctum that co-localise in all three channels. Scale bar represents 5µm.

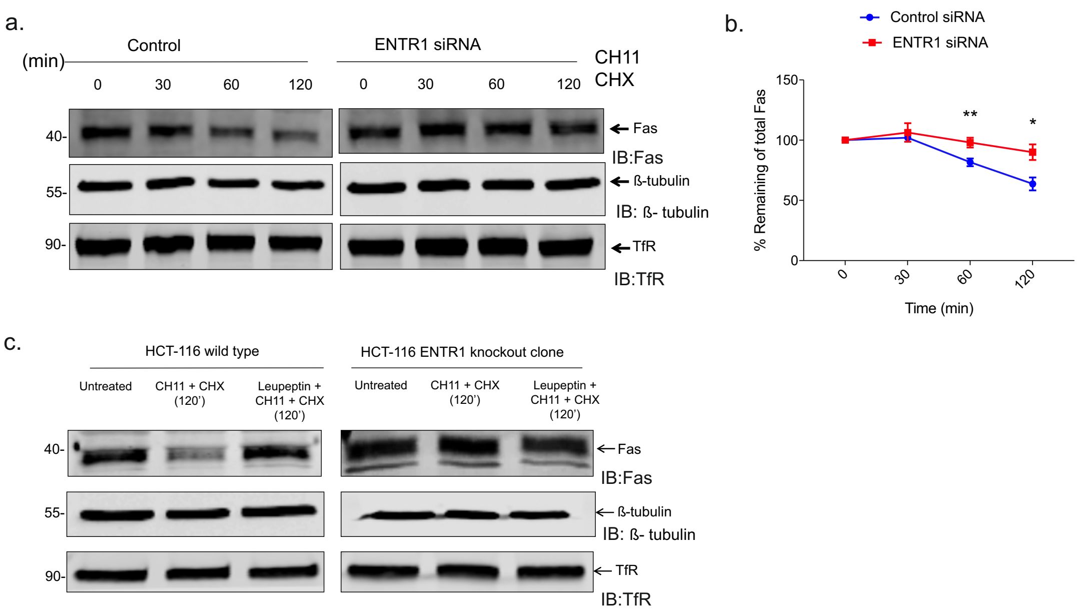

Supplementary Figure 5: Degradation kinetics of endogenous Fas (a) Immunoblot analysis of the kinetics of degradation of endogenous Fas in HCT-116 cells in control or ENTR-1 siRNA transfected cells treated with agonistic anti-Fas (CH-11) antibody (500 ng/ml) and cycloheximide (150µg/ml) for the indicated time points. (b) Relative receptor abundance expressed as a percentage where 0 min is 100% in control or ENTR1 siRNA treated HCT-116 cells, respectively., normalized to ß-tubulin expression. Data were collected from three independent experiments (n=3) and analysed by multiple comparisons test, $*p<0.05$, error bars represent ± SEM. (c) Immunoblot analysis of the kinetics of degradation of endogenous Fas in wild-type and ENTR1 knockout HCT-116 cells treated with agonistic anti-Fas (CH-11) (500 ng/ml) and cycloheximide (150µg/ml) and with or without the pre-treatment with Leupeptin (100 nM) at the indicated time points. ß-tubulin was used as a loading control and transferrin receptor (TfR) was used as a control recycling receptor

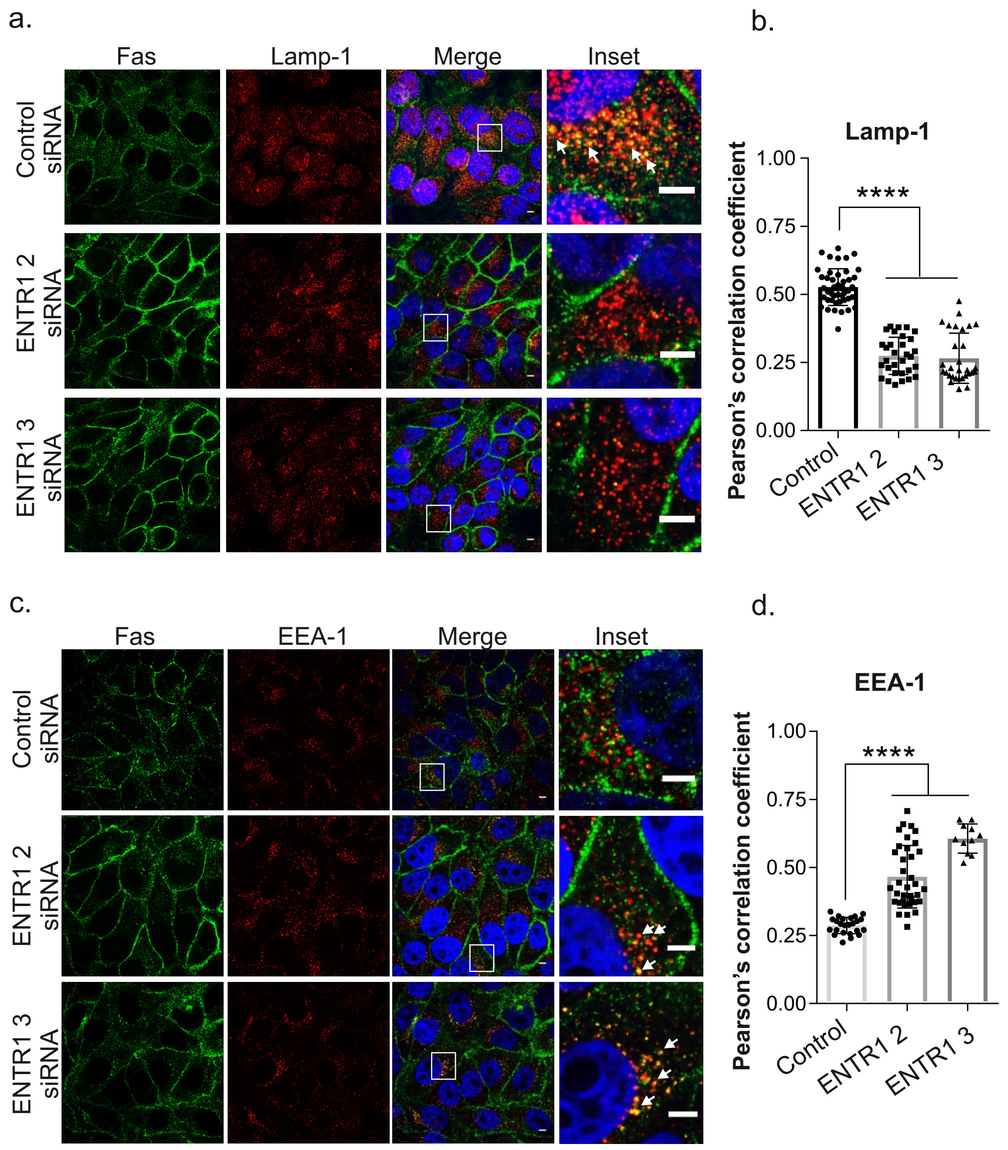

Supplementary Figure 6: Depletion of ENTR1 traps Fas receptors into early endosomes delaying their entry into late endosome/lysosomes. HeLa cells treated with control or ENTR1 no.2 and 3 siRNA were subjected to bath application of non-agonistic anti-Fas (DX-2) antibody (1µg/ml) in the presence of Leupeptin (100nM). After 6 hours of antibody feeding, cells were fixed and stained for endosomal markers such as Lamp-1 (a) and EEA-1 (c). Arrows indicate co-localisation between Fas and Lamp-1 (a) or EEA-1 (c). Images were acquired using a confocal microscope. Scale bars represent 5µm (a,c). Quantification of Pearson's correlation coefficient observed between Fas receptors and Lamp-1 (b) or EEA-1 (d) under different treatment conditions. Co-localisation was analysed using JACoP (*Just another co-localisation plugin*) in ImageJ. At least 300 cells were analysed from three independent experiments. One-way ANOVA was performed, ****$p<0.0001$, error bars represent ±s.e.m.

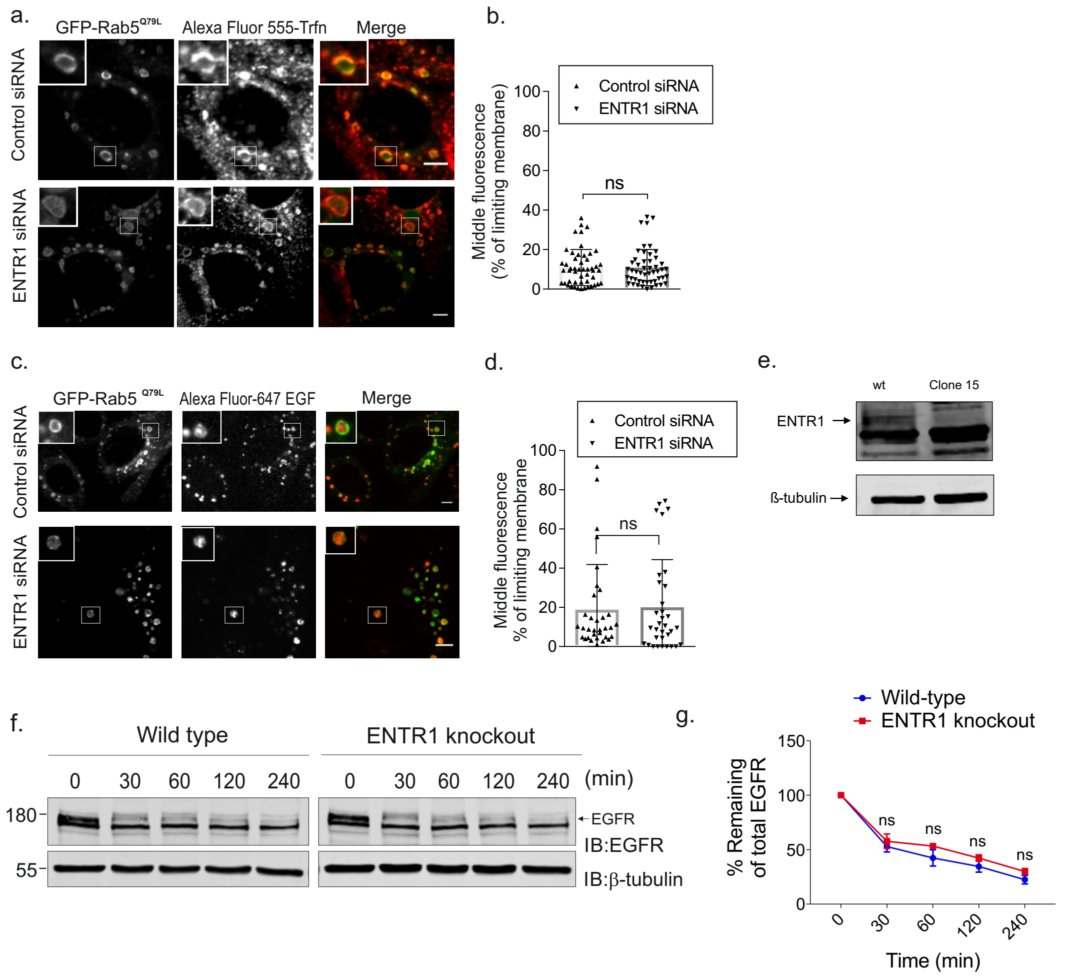

Supplementary Figure 7: Depletion of ENTR1 does not affect intraluminal sorting of Transferrin and EGF receptors (a) HeLa cells transfected with Rab5 Q79L and treated with control or ENTR1 no. 3 siRNA were stimulated with Alexa 555 conjugated anti-transferrin for 1 hour in the presence of Leupeptin (100nM). Inset panel shows representative enlarged endosomes showing internalized transferrin localised on the limiting membrane in both control and ENTR1 siRNA treated cells. (b) Quantification of the mean fluorescence intensity of transferrin inside the intraluminal vesicle expressed as a percentage. Data was collected form at least 100 endosomes from three independent experiments. Student's t-test, ns- not significant, p=0.8297, error bars represent ±s.e.m (c) Intraluminal sorting analysis for Alexa 647 conjugated EGF in control and ENTR1 no.3 siRNA treated HeLa cells expressing Rab5 Q79L transiently. Cells were stimulated for 1 hour with conjugated EGF in the presence of Leupeptin (100nM). Images were acquired using a confocal microscope. Representative enlarged endosomes are highlighted in insets. Scale bar represents 5µm. (d) Quantification of the mean fluorescence of EGF entering into ILVs. Student's t test was performed for more than 100 cells from three independent experiment, ns-not significant, p=0.9870, error bars represent ±s.e.m (e) Validation of the ENTR1 knock-out in HeLa cell line (clone 15) by Western Blotting. ß-tubulin shown as a loading control. (f) Immunoblot analysis of the kinetics of degradation of endogenous EGFR activated by EGF (100 ng/ml) for the indicated time points in HeLa WT and HeLa ENTR1 knock-out cell line (clone 15). ß-tubulin was used as a loading control. (g) Relative receptor abundance expressed as a percentage where 0

min is 100% in wild-type or ENTR1 knock-out HeLa cells, respectively. Data were collected from three independent experiments (n=3) and analysed by multiple comparisons test, *$p<0.05$, error bars represent ± SEM.

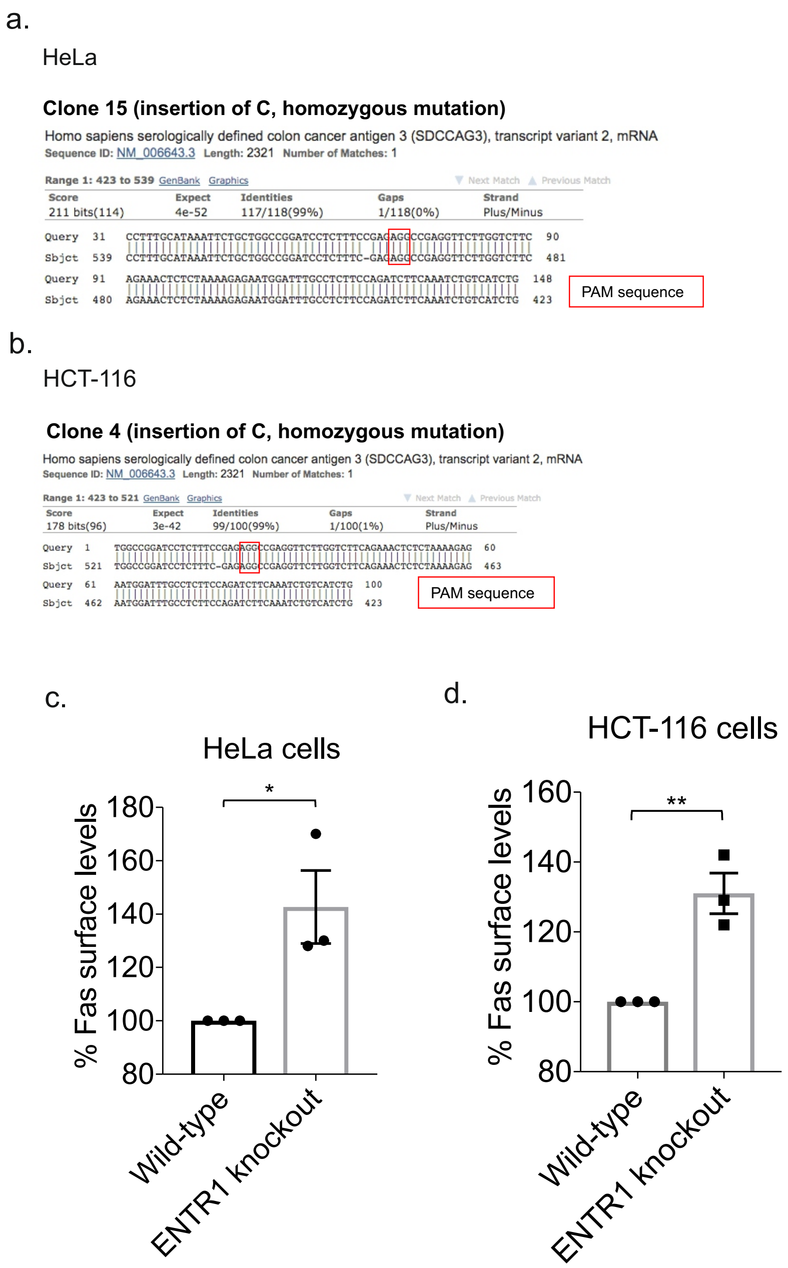

Supplementary Figure 8: ENTR1 knockout cell lines generated by CRISPR-Cas9 show increased surface Fas expression. (a) Validation of the ENTR1 knock-out in HeLa cell line (clone 15) by sequence analysis of the gRNA target region (b) Validation of the ENTR1 knockout in HCT-116 cell line (clone 4) by sequence analysis of the gRNA target region. PAM sequence is indicated in red. (c,d) Flow cytometry analysis of surface levels of the Fas receptor in ENTR1 knockout compared to wild-type HeLa cells (c) or wildtype HCT-116 cells (d), data from three independent experiments, n=3, *$p<0.05$, **$p<0.01$, student t-test was performed, error bars represent ±s.e.m. Live cells were stained with anti-Fas CH11 primary antibody followed by Alexa 488 conjugated secondary antibody and analysed in the BD LSRII flow cytometer.

a.

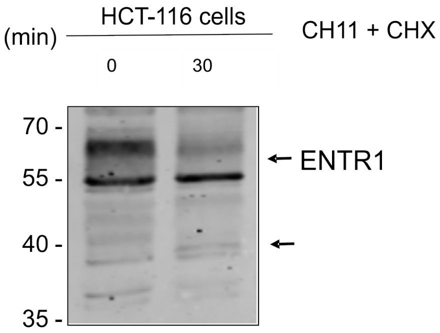

b.

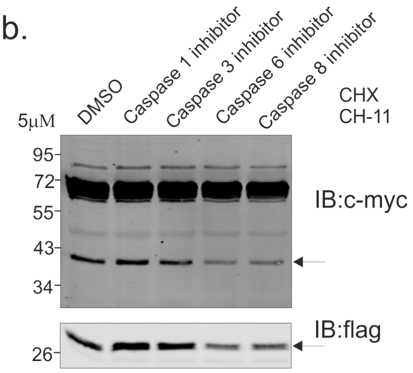

c.

Sequence	Modifications	Residues	Mass	Proteins	Gene Names	Protein Names	Charges	PEP	Score	Delta score
SRVSPASPAGSPSADFAVHGESLGDR	Unmodified	237-262	2553.22	Q96C92	ENTR1	Endosome associated trafficking regulator 1	3	0.00017	139.79	83.64
SRVSPASPAGSPSADFAVHGESLGDR	Phospho (STY)	237-262	2633.19	Q96C92	ENTR1	Endosome associated trafficking regulator 1	3	0.02887	91.123	49.831
SRVSPASPAGSPSADFAVHGESLGDR	2 Phospho (STY)	237-262	2713.15	Q96C92	ENTR1	Endosome associated trafficking regulator 1	3	0.00524	117.52	69.598

d.

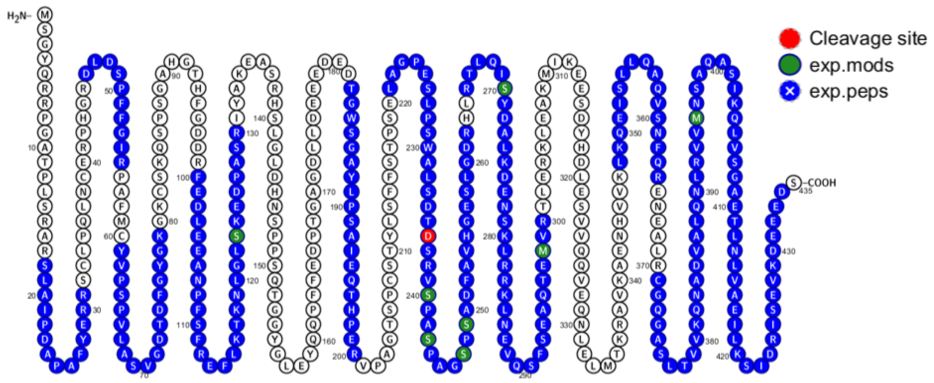

Supplementary Figure 9: (a) Fas-mediated caspase cleavage of endogenous ENTR-1 upon treatment with anti-Fas (CH-11) antibody and CHX at the indicated time points. Arrow indicates the cleaved fragments (b) Cleavage of overexpressed myc-ENTR1-flag is reduced upon activation of Fas-mediated apoptosis with anti-Fas (CH11, 200 ng/ml) and cycloheximide (2.5 µg/ml) and in the presence of caspase 6 (Z-VEID-FMK) and caspase 8 (Z-IETD-FMK) inhibitors but not of caspase 1 (Z-YVAD-FMK) or caspase 3 inhibitors (Z-DEVD-FMK) (c) Mapping the caspase cleavage site on ENTR1 using mass spectrometry. Peptide sequences identified from ENTR1-Flag immunoprecipitations after tryptic digestion. The N-terminus of each peptide was generated by cleavage at aspartate 236 (d) Sequence coverage of ENTR1 using mass spectrometry with the cleavage site indicated in red; exp.mods: experimental modifications, exp.peps: experimental peptides.

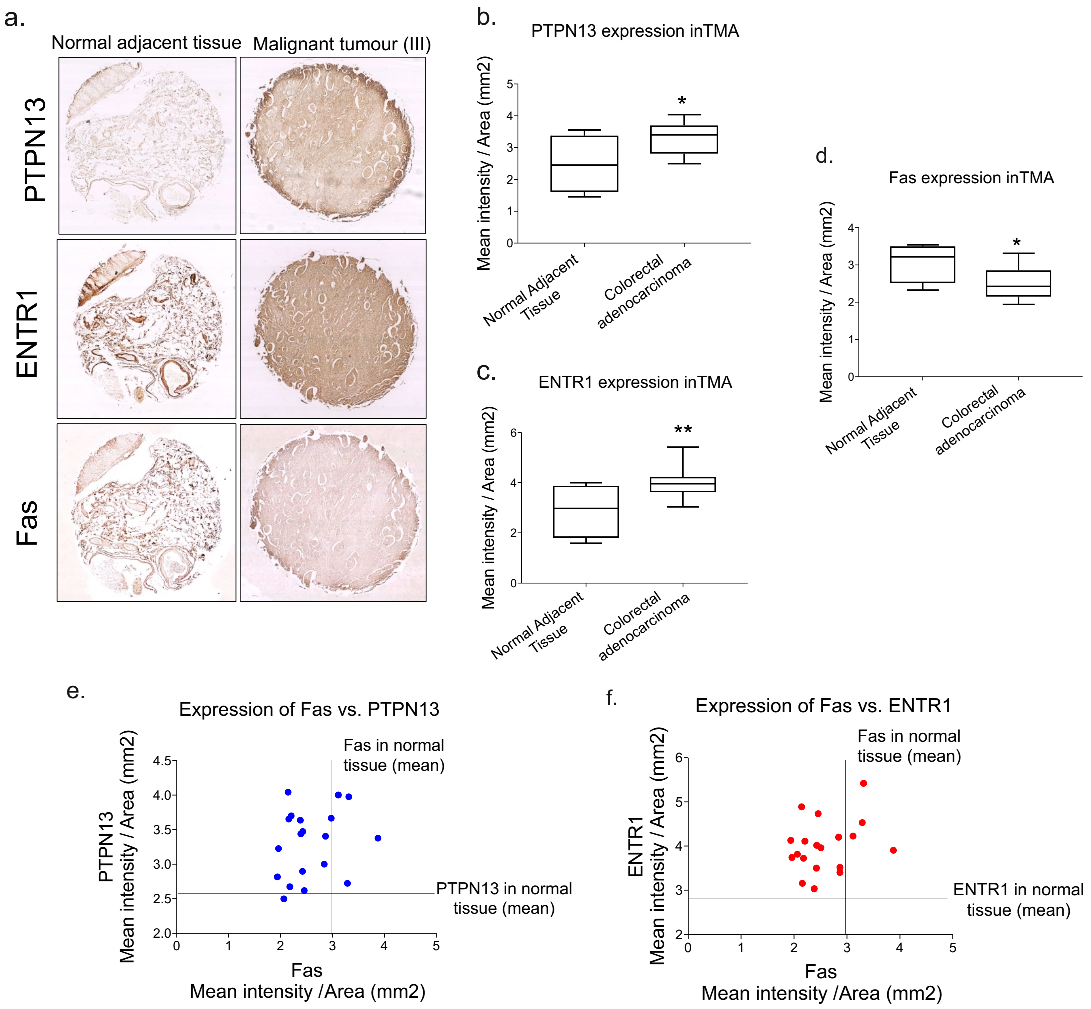

Supplementary Figure 10: PTPN13/ENTR1 expression is upregulated and Fas expression is downregulated in colon cancer. (a) Examples of immunohistochemical staining of PTPN13, ENTR1 and Fas in a colon cancer TMA (CO242b, Biomax US) indicating weak expression of the markers in normal adjacent colon tissue and a moderate/strong staining for PTPN13 and ENTR1 in the colon cancer histocore whereas weak staining for Fas in the same colon cancer histocore. (b-d) Box and whiskers plots displaying the expression of PTPN13 (b), ENTR1 (c) and Fas (d) in colorectal cancer TMAs. Expression of the markers in normal adjacent tissue is compared to colorectal adenocarcinoma histocores in terms of mean intensity of staining in each core / area of the core (mm2). Data was collected from three independent experiments (n=3) comparing 4 normal adjacent tissue samples to 19 colorectal adenocarcinoma histocores and statistical significance was analysed by t-test. Box represents the interquartile range and the middle line represents the median, whiskers represent minimum to maximum values. (e,f) Dot plots displaying the expression of Fas and either PTPN13 (e) or ENTR1 (f) in colorectal adenocarcinoma samples (n=19). p-value (ENTR1 vs Fas) = 0,00587 (**); p-value (PTPN13 vs Fas) = 0,01970 (*) were calculated by one-tailed proportion test (z). Internal axis corresponds to the mean expression of the corresponding markers in normal adjacent colon tissue. Imaging data were collected from three independent experiments (n=3).

Figure 1 c.

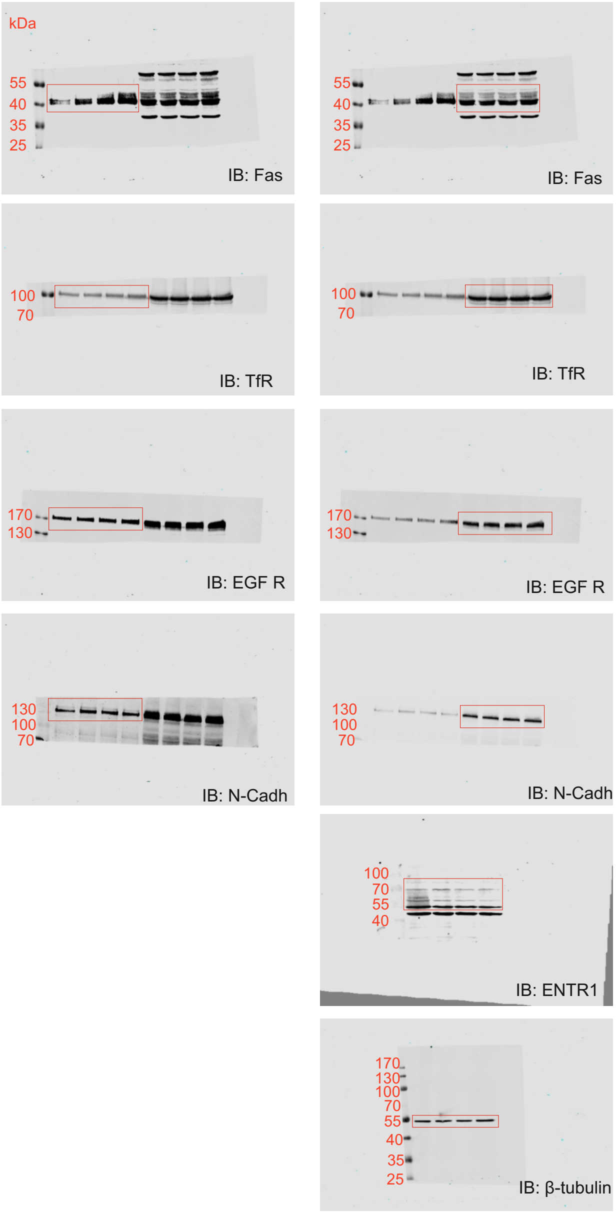

Figure 1 e.

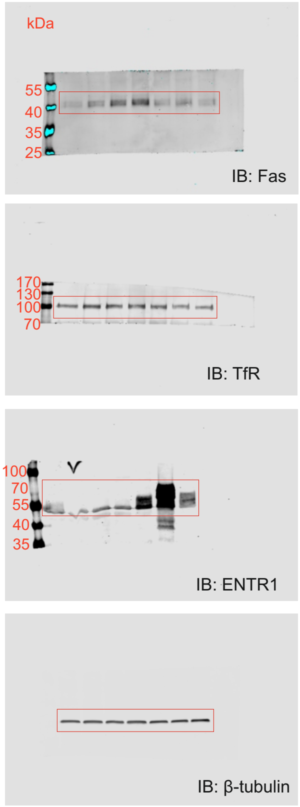

Figure 2 c.

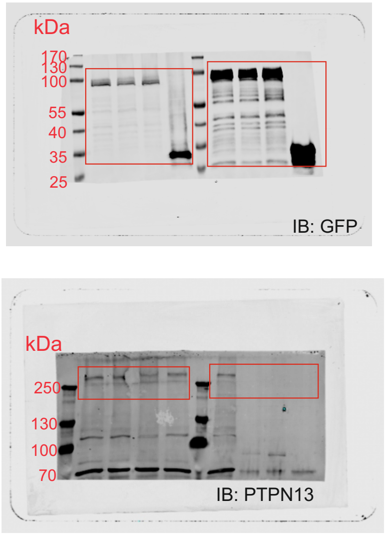

Figure 4 a.

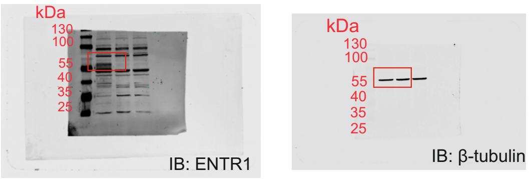

Figure 4 b.

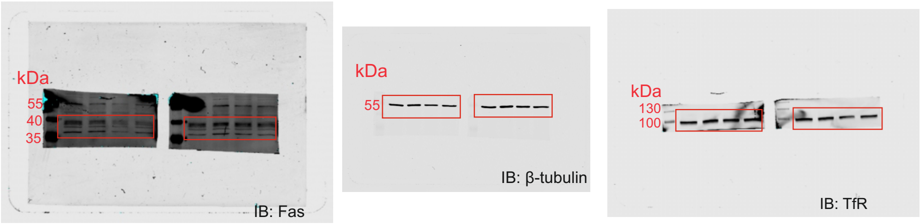

Figure 6 a.

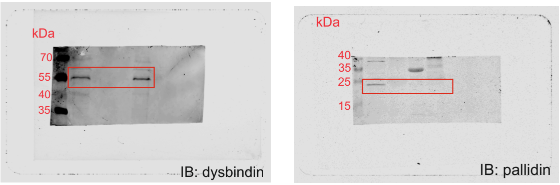

Figure 6 b.

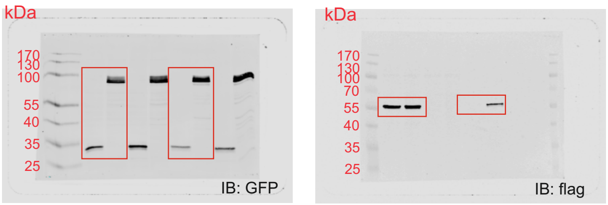

Figure 6 d.

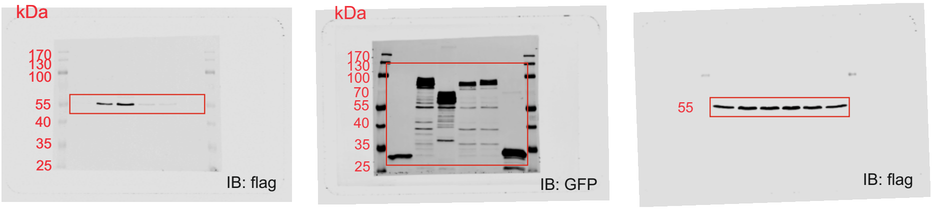

Figure 7 c.

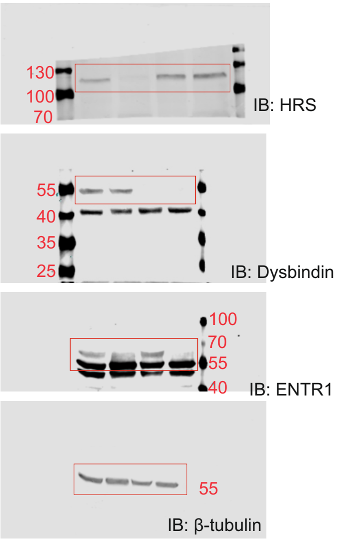

Figure 8 b.

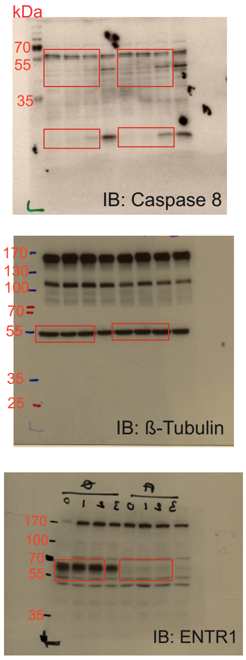

Figure 9 a.

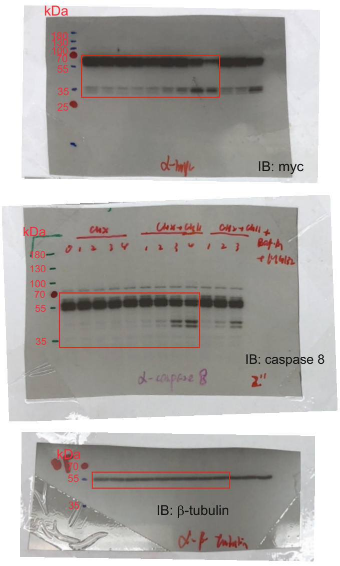

Figure 9 b.

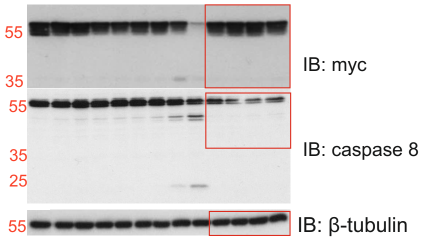

Figure 9 d.

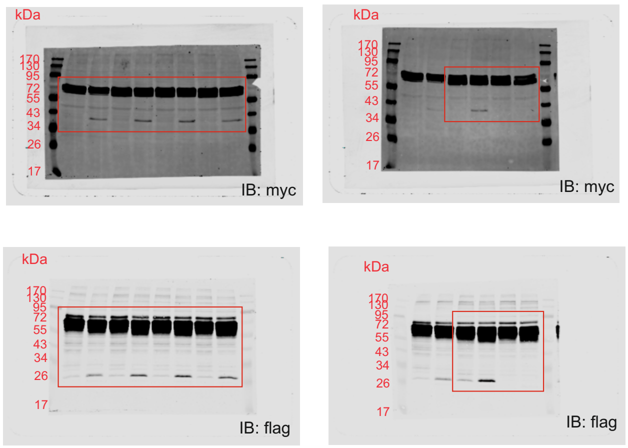

Figure 9 e.

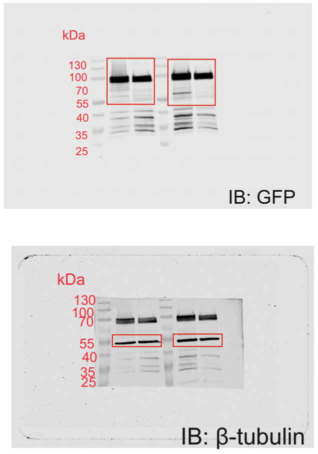

Supplementary Figure 2 c.

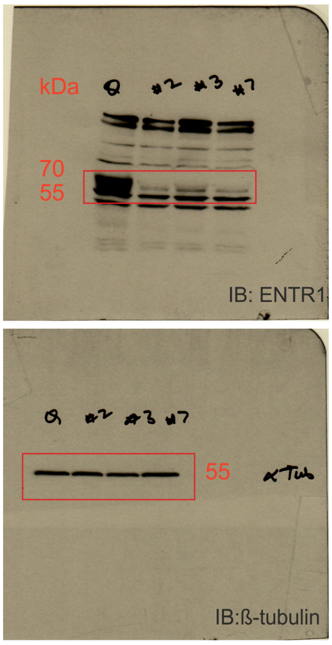

Supplementary Figure 3 a.

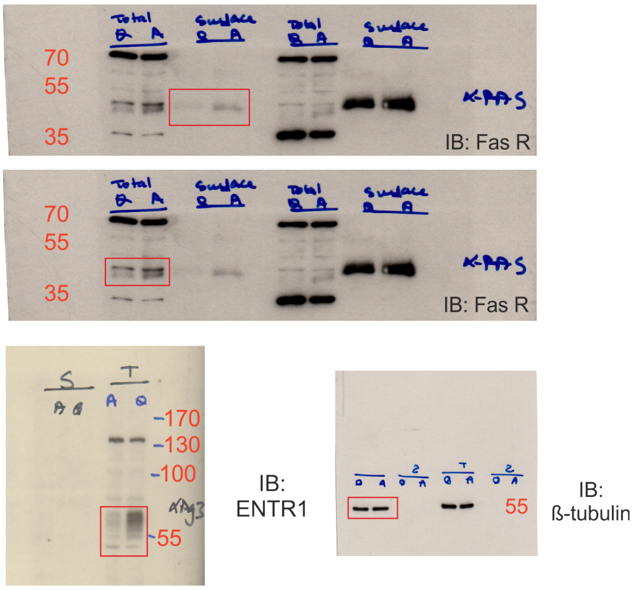

Supplementary Figure S3 d.

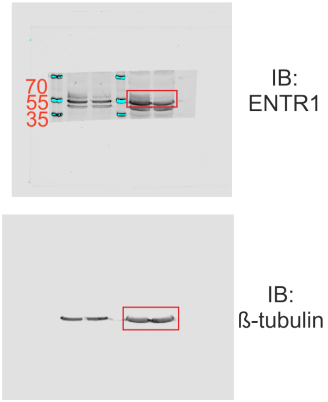

Supplementary Figure 3 e.

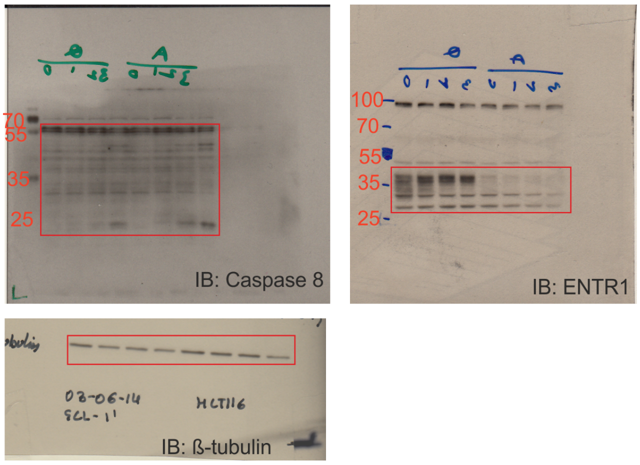

Supplementary Figure 5 f.

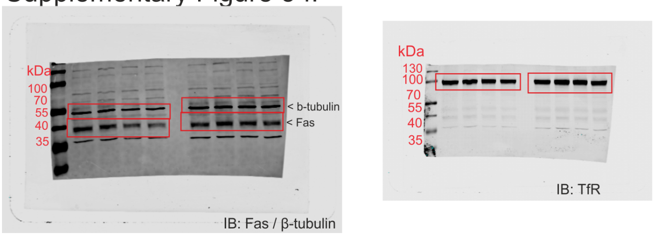

Supplementary Figure 5 h.

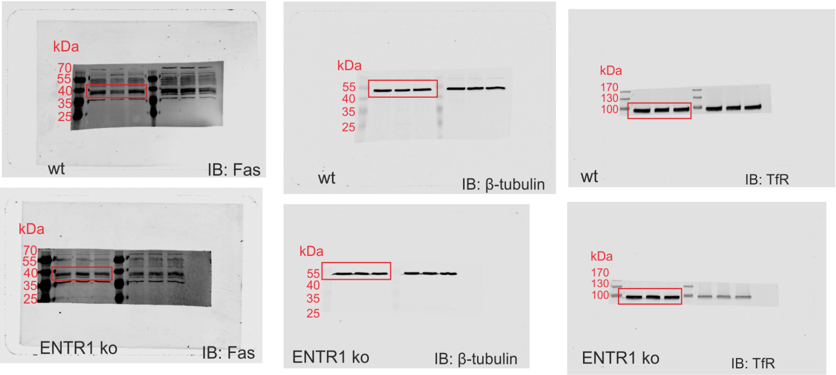

Supplementary Figure 7 e.

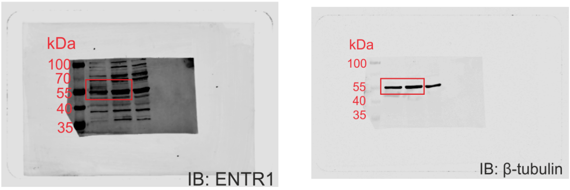

Supplementary Figure 7 f.

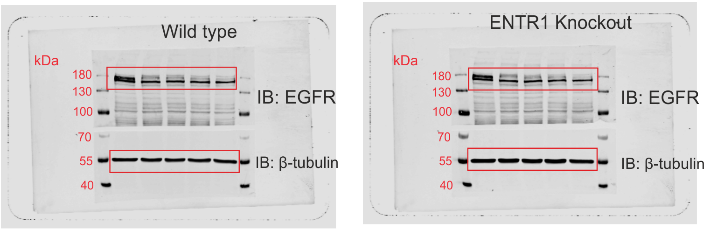

Supplementary Figure 9 a.

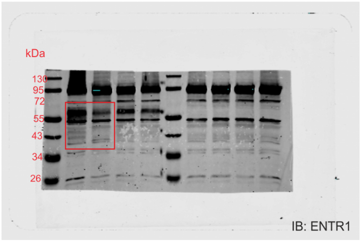

Supplementary Figure 9 b.

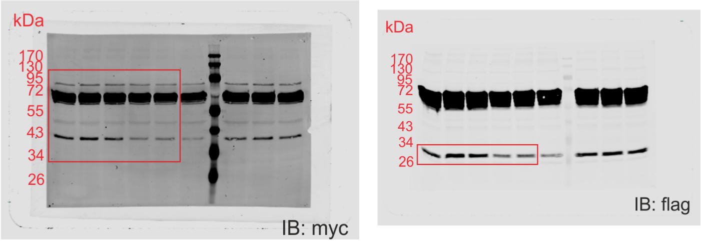

Supplementary Figure 11. Full-blots used to prepare figures. Shown are uncropped scans from films, images taken with the LI-COR Odyssey Sa. System. Red rectangles indicate the selected images for the final figures.
