# Supplementary material for: Apoptotic signalling targets the post-endocytic sorting machinery of the death receptor Fas/CD95
Source: Nat Commun. 2019 Jul 15;10:3105. doi: 10.1038/s41467-019-11025-y (PMC6629679; doi:10.1038/s41467-019-11025-y)
Supplement: Supplementary file 2 — Reporting Summary [file 41467_2019_11025_MOESM2_ESM.pdf]

nature research

Corresponding author(s): NCOMMS-16-26533C

Last updated by author(s): May 31, 2019

Reporting Summary

Nature Research wishes to improve the reproducibility of the work that we publish. This form provides structure for consistency and transparency in reporting. For further information on Nature Research policies, see Authors & Referees and the Editorial Policy Checklist.

Statistics

For all statistical analyses, confirm that the following items are present in the figure legend, table legend, main text, or Methods section.

n/a	Confirmed	
☐	☒	The exact sample size (*n*) for each experimental group/condition, given as a discrete number and unit of measurement
☐	☒	A statement on whether measurements were taken from distinct samples or whether the same sample was measured repeatedly
☐	☒	The statistical test(s) used AND whether they are one- or two-sided<br>*Only common tests should be described solely by name; describe more complex techniques in the Methods section.*
☒	☐	A description of all covariates tested
☒	☐	A description of any assumptions or corrections, such as tests of normality and adjustment for multiple comparisons
☐	☒	A full description of the statistical parameters including central tendency (e.g. means) or other basic estimates (e.g. regression coefficient) AND variation (e.g. standard deviation) or associated estimates of uncertainty (e.g. confidence intervals)
☒	☐	For null hypothesis testing, the test statistic (e.g. F, t, r) with confidence intervals, effect sizes, degrees of freedom and P value noted<br>*Give P values as exact values whenever suitable.*
☒	☐	For Bayesian analysis, information on the choice of priors and Markov chain Monte Carlo settings
☒	☐	For hierarchical and complex designs, identification of the appropriate level for tests and full reporting of outcomes
☒	☐	Estimates of effect sizes (e.g. Cohen's *d*, Pearson's *r*), indicating how they were calculated

Our web collection on statistics for biologists contains articles on many of the points above.

Software and code

Policy information about availability of computer code

Data collection	no software used
Data analysis	Image J 1.52 and GraphPad Prism 7 (7.01)

For manuscripts utilizing custom algorithms or software that are central to the research but not yet described in published literature, software must be made available to editors/reviewers. We strongly encourage code deposition in a community repository (e.g. GitHub). See the Nature Research guidelines for submitting code & software for further information.

Data

Policy information about availability of data

All manuscripts must include a data availability statement. This statement should provide the following information, where applicable:

- Accession codes, unique identifiers, or web links for publicly available datasets
- A list of figures that have associated raw data
- A description of any restrictions on data availability

The data that support the findings of this study are available from the corresponding author upon reasonable request.

Field-specific reporting

Please select the one below that is the best fit for your research. If you are not sure, read the appropriate sections before making your selection.

☒ Life sciences ☐ Behavioural & social sciences ☐ Ecological, evolutionary & environmental sciences

For a reference copy of the document with all sections, see nature.com/documents/nr-reporting-summary-flat.pdf

Life sciences study design

All studies must disclose on these points even when the disclosure is negative.

Sample size	To calcualte the standard error means at least three independent experiments were performed, the sample size is indicated for each experiment
Data exclusions	no data were excluded
Replication	All results have been replicated
Randomization	NA
Blinding	Blinding was not necessary as data were quantified

Reporting for specific materials, systems and methods

We require information from authors about some types of materials, experimental systems and methods used in many studies. Here, indicate whether each material, system or method listed is relevant to your study. If you are not sure if a list item applies to your research, read the appropriate section before selecting a response.

Materials & experimental systems

n/a	Involved in the study
☐	☒ Antibodies
☐	☒ Eukaryotic cell lines
☒	☐ Palaeontology
☒	☐ Animals and other organisms
☒	☐ Human research participants
☒	☐ Clinical data

Methods

n/a	Involved in the study
☒	☐ ChIP-seq
☐	☒ Flow cytometry
☒	☐ MRI-based neuroimaging

Antibodies

Antibodies used	ENTR1 (15969-1-AP, 1:300 for IF) Proteintech, Manchester, UK; ENTR1 (HPA029303, 1:250 for IB), ß-tubulin (T4062, 1:5000 for IB), Anti-Flag M2 (1:1000 for IB) Sigma-Aldrich, England; Fas (C-20, 1:500 for IB), N-Cadherin (13A9, 1:500 for IB), rabbit anti-c-Myc (A-14, 1:100 for IB), rabbit anti-Fas (C-20, 1:500 IB), rabbit anti-FAP1 (H-300, 1:500 for WB), goat anti-EEA-1 (N-19, 1:100 for IF) Santa Cruz (USA). Anti-Fas clone CH-11 (1:500 for IF and FC) Millipore and anti-Fas clone DX-2 (1:500 for FC) Biolegend (USA). CD71 (D7G9X) XP (1:2000 for IB), EGF (2232, 1:1000 for IB), rabbit anti-EEA-1 (C45B10, 1:300 for IF), anti-HA (6E2, 1:1000 for IB), anti-Caspase 8 (1C12, 1:1000 for IB), anti-cleaved caspase 3 (Asp175, 1:400 for IF), anti-HRS (14346, 1:1000 for IB) Cell signalling (USA). Anti-Dysbindin (ab124967, 1:1000 for IB), mouse anti-pallidin (ab169037, 1:500 for IB), rabbit anti-Lamp-1 (1: 800 for IF) Abcam (Cambridge, UK). Alexa 594 conjugated. Transferrin Invitrogen and anti-CD261/TRAIL-R1 FITC conjugated (clone DR-4-02) Life technologies. Rabbit anti-PTPN13 (NB100-56139, 1:500 for IF; 1:1000 for IHC) from Novus Bio. Rabbit anti-GFP was a kind gift from Andrew Peden, University of Sheffield (1:2000 for IB)
Validation	ENTR1 and PTPN13 antibodies have been validated with corresponding siRNA experiments using western-blotting and immunofluorescence analysis as a readout

Eukaryotic cell lines

Policy information about cell lines

Cell line source(s)	HeLa and HCT116 cells are from Sigma- Aldrich
Authentication	Visual inspection
Mycoplasma contamination	Cell lines were tested for mycoplasma contamination every six months and test have been negative
Commonly misidentified lines (See ICLAC register)	N.A.

Flow Cytometry

Plots

Confirm that:

☒ The axis labels state the marker and fluorochrome used (e.g. CD4-FITC).

☒ The axis scales are clearly visible. Include numbers along axes only for bottom left plot of group (a 'group' is an analysis of identical markers).

☒ All plots are contour plots with outliers or pseudocolor plots.

☒ A numerical value for number of cells or percentage (with statistics) is provided.

Methodology

Sample preparation	Cells were detached from the culture dish with trypsin-EDTA for 5 min and washed once with 1% BSA/PBS and incubated with either Alexa conjugated or unconjugated primary antibodies for 1 hour at 4°C. After labelling with secondary antibody, cells were analysed immediately by BD LSRII™ or BD FACSCalibur™ flow cytometers (BD Biosciences). Secondary antibody alone was used as the negative control for determining specificity of the signal.
Instrument	BD LSRII™ or BD FACSCalibur™ flow cytometers (BD Biosciences)
Software	FLOWJO
Cell population abundance	The percentage of GFP-positive cells was between 30 and 50%
Gating strategy	Gate boundaries for GFP positive cells were set 80-230 FSC-A (x1000) and 30-180 SSC-A (x1000)

☐ Tick this box to confirm that a figure exemplifying the gating strategy is provided in the Supplementary Information.
